# Synergistic enhancement of magneto-optical response in cobalt-based metasurfaces via plasmonic, lattice, and cavity modes

**DOI:** 10.1515/nanoph-2025-0495

**Published:** 2025-12-09

**Authors:** Alberto Santonocito, Alessio Gabbani, Barbara Patrizi, Guido Toci, Francesco Pineider

**Affiliations:** Dipartimento di Chimica e Chimica Industriale, Università di Pisa, Via Moruzzi 13, 56124, Pisa, Italy; Consiglio Nazionale delle Ricerche (CNR), Istituto Nazionale di Ottica (INO), Via Madonna del Piano 10, 50019, Sesto Fiorentino (FI), Italy; Dipartimento di Chimica “Ugo Schiff”, Università di Firenze, Via della Lastruccia 13, 50019, Sesto Fiorentino (FI), Italy

**Keywords:** tunable metasurfaces, Gires–Tournois interferometers, magnetoplasmonics

## Abstract

Static metasurfaces offer precise control over light but lack reconfigurability, limiting their use in dynamic applications. Introducing tunability via external stimuli, such as magnetic fields, enables active control of their optical response, broadening their functionality. In this computational study, we present the design of a metal–dielectric–metal magnetoplasmonic metasurface with improved magnetic field tunability, surpassing the magneto-optical response of unstructured ferromagnetic materials. This improvement arises from the synergistic effect of localized plasmon excitation, surface lattice resonance, and Fabry–Pérot cavity modes. The design approach presented here consists in matching the characteristic resonance frequencies of the three phenomena by iteratively adjusting the structural parameters of the metasurface: nanostructure size, lattice period, and cavity layer thickness. This optimization led to a substantial enhancement in the reflectance modulation induced by an external magnetic field, with the overall contrast exceeding that of an unstructured cavity by more than an order of magnitude across various regions of the visible to near-infrared spectrum, under relatively low magnetic fields. This unique capability makes the system a promising tool for magnetic field-sensitive optical modulation of reflected light intensity, with potential applications as a laser amplitude modulator.

## 1 Introduction

Metasurfaces constitute an emerging class of engineered materials that exhibit unique electromagnetic wave manipulation capabilities. These structures consist of two-dimensional periodic arrays of subwavelength scatters, commonly known as meta-atoms, arranged in distinct patterns. The ability of metasurfaces to precisely control the phase, amplitude, and polarization of incident electromagnetic waves has generated considerable interest within the scientific community, paving the way for novel applications in optics and photonics [1], [2]. At the nanoscale, these surfaces interact with incoming light, inducing absorption or coherent scattering phenomena, thus altering the properties of the reflected or transmitted light. This flexibility has stimulated the exploration of diverse optical applications due to the advantages of metasurfaces over traditional optical components notably in terms of efficiency, affordability, and compactness.

Although static metasurfaces have been extensively studied, the practical realization of their full potential in optical devices would be strongly enhanced by the development of reconfigurable metasurfaces, featuring the capability to dynamically adjust their optical response in real-time [3], [4]. Reconfigurable metasurfaces have emerged as a versatile technology with significant potential across a variety of optical applications. With dynamic control over phase, amplitude, and polarization of light, these metasurfaces are particularly valuable in several fields:

1. High-speed optical communication systems, where they can optimize data transmission and reduce signal distortion;
2. Optical imaging, where they offer the potential to replace traditional components like lenses and mirrors, providing adaptive and real-time control over parameters such as focal length, aberrations, and optical distortions (*i.e.*, defocus and astigmatism) [5], [6];
3. Sensing applications, where they can enhance sensitivity and resolution by adapting to environmental changes or specific target molecules, optimizing both spatial resolution and contrast;
4. Security and defense, such as adaptive cloaking, where they alter light–object interactions to reduce detection by optical or radar sensors;
5. Consumer electronics, where they can be integrated into devices like smart glasses or augmented reality headsets, offering real-time optical adjustments for improved user experience.

A promising tool that could revolutionize the field of reconfigurable metasurfaces is the integration of magnetic field control. By utilizing magnetic fields, the metasurfaces’ tunability could be achieved with a noncontact, rapid, and reversible modulation of the optical response, without the need for mechanical moving parts. This kind of modulation does not need physical contact with the sample such as electrical modulation. Moreover, magnetic field application does not cause the degradation of the material after multiple cycles of modulation as more often occurs in thermal modulation or all optical modulation, which requires irradiation with high power lasers. This approach offers substantial improvements in speed, precision, and versatility, unlocking new possibilities for a wide range of optical applications [7], [8], [9]. We note that the technology for generating intense and rapidly modulated magnetic fields on submicrometer spatial scale is already available from the magnetic hard disks for data storage, where writing heads can generate field strengths up to about 1 T, switched at GHz bandwidth on spatial scales of the order of 100 nm [10]. Despite a few works on magnetically tunable metasurfaces have been reported already, most of the work have been done in the THz range [11], while only a few cases are reported in the visible or near infrared spectral range [12], [13]. A promising approach toward the improvement of magnetic field tunability of the optical response is certainly represented by the excitation of plasmonic resonance in metallic nanostructures, a phenomenon exploited by researchers in magnetoplasmonics over the last two decades, and which is still a vibrant and active field of research [14], [15], [16], [17].

In this work, we chose cobalt (Co) as active magneto-optical material for the metasurfaces, a ferromagnetic metal with a high Curie temperature (1,388 K) and strong magneto-crystalline anisotropy, currently representing together with nickel (Ni) the state-of-the-art of magnetoplasmonic materials [14], [18], being it widely employed for magnetoplasmonics [15], [19] in combination with noble metals. In our approach, Co nanostructures are arranged in periodic pattern exploiting surface lattice resonance (SLR), which proved to significantly enhance magnetoplasmonic modulation [20], particularly through multilayered metal–dielectric–metal nanostructures [13], [21], [22], [23], [24]. In this context, Fabry–Pérot cavities could enhance light–matter interactions, providing high electromagnetic field confinement and optical modulation achievable by adjusting the cavity length [25], [26], [27]; on the other hand, they have been rarely exploited for magnetoplasmonics, and most of the reports are in the THz spectral range [28]. We propose the synergistic exploitation of these interactions to amplify the effects of magnetic field modulation, enabling more efficient and precise control of light propagation. In this study, we employ numerical simulations to design metasurfaces with metal–dielectric–metal structure, consisting of a metallic bulk substrate coated with a thin layer of Co, a dielectric spacer, and a periodic array of Co nanostructures atop the spacer. Our simulated metasurfaces are based on a specific modification of the Fabry–Pérot interferometer, known as the Gires–Tournois (GT) interferometer. We begin our investigation on how an external magnetic field influences the magneto-optical response of GT metasurfaces. We conducted an extensive parametric study of the behavior of the magneto-optical interactions by varying the metasurface structural parameters, and we devised structures that exhibit enhanced magneto-optical response. We observed high reflection contrast and significant phase rotation variations when GT metasurfaces interacted with Right Circularly Polarized (RCP) and Left Circularly Polarized (LCP) light in the presence of an external magnetic field. We propose to exploit this effect to realize a magnetically tunable intensity modulator to offer precise control over reflected light intensity and polarization.

This work establishes the basis for the design of magnetic field tunable metasurfaces exploiting metal–dielectric–metal cavities, which provide concrete criteria to finally push magnetoplasmonic modulation toward real-life tunable miniaturized nano-devices such as optical switches, lenses, or sensors.

## 2 Design and simulation methodology

In our study, we employed a Finite Element Analysis [29] (FEA) modeling environment (COMSOL Multiphysics [30]). The structures that we propose are based on Co, Al, and SiO_2_. The simulations employ the dielectric functions for Co [31] and Al [32], while the optical properties of silica (SiO_2_) are described by its refractive index [33]. We consider planar structures extending in the *x*–*y* plane. The incident electromagnetic wave propagates in the *z* direction; the electric field associated to the electromagnetic wave has both *x* and *y* components (*E*_*x*_ and *E*_*y*_), and it has the general form: $E=E_{x}\cos\left(\omega t\right)\hat{x}+iE_{y}\sin\left(\omega t\right)\hat{y}$ . We set *E*_*x*_ = 1 V/m and *E*_*y*_ = ±1 V/m. Further details regarding the definitions and conventions used for the employed physical quantities can be found in the Supplementary Information.

In the presence of a magnetic field parallel to the electromagnetic wave *k*-vector, reflective ferromagnetic materials exhibit the polar Magneto-Optical Kerr Effect (*p*-MOKE), which modifies the polarization and intensity of reflected light. This effect arises from the off-diagonal components of the material permittivity tensor, influenced by the external magnetic field and the material magnetization (further details on *p*-MOKE can be found in the Supplementary Information).

In our geometry, the dielectric permittivity *ε* of Co can be expressed as follows:

(1) $$\varepsilon =\left(\begin{array}{ccc} \varepsilon_{xx} & \varepsilon_{xy} & 0\\ \varepsilon_{yx} & \varepsilon_{yy} & 0\\ 0 & 0 & \varepsilon_{zz} \end{array}\right)$$

where $\varepsilon_{xx}=\varepsilon_{yy}=\varepsilon_{zz}=\varepsilon_{xx}^{\prime }-i\varepsilon_{xx}^{\prime \prime }$ and $\varepsilon_{xy}=-\varepsilon_{yx}=\varepsilon_{xy}^{\prime }-i\varepsilon_{xy}^{\prime \prime }$ ; $\varepsilon_{xx}^{\prime }$ ( $\varepsilon_{xy}^{\prime }$ ) and $\varepsilon_{xx}^{\prime \prime }$ ( $\varepsilon_{xy}^{\prime \prime }$ ) are the real and the imaginary parts of the complex diagonal (off-diagonal) elements of the permittivity tensor. The values of the off-diagonal terms were taken from reference [18].

The resulting *p*-MOKE is characterized by polarization plane rotation and ellipticity upon reflection of Linearly Polarized (LP) light, which can be described by decomposing LP light into RCP and LCP components, as exemplified in Figure 1S in Supplementary Information.

**Figure 1:**
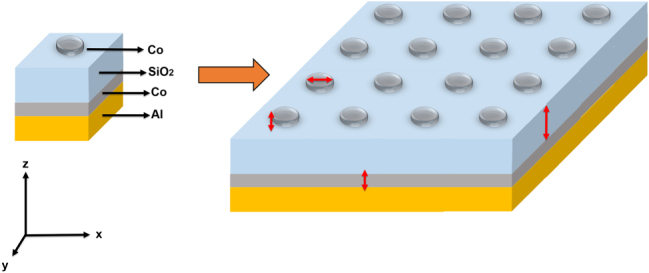
Architecture of the simulated metasurfaces. Unit cell of the simulated system and a pictorial representation of the entire simulated metasurface showing the periodic arrangement of the unit cells.

The structure of the simulated metasurfaces (Figure 1) consists of two main components: the multilayered substrate and the ferromagnetic meta-atoms. The substrate structure includes bulk Al reflector at the bottom, followed by a thin ferromagnetic layer (Co) and a layer of dielectric (SiO_2_). A disk, composed of the same ferromagnetic material (Co) as the coating and supporting localized surface plasmon resonance, is positioned atop the SiO_2_ layer. The unit cell of the metasurface is surmounted by air situated above the disk.

Figure 1 illustrates the unit cell and the full GT metasurface structure, along with the relevant size parameters of the calculations (red double-headed arrows).

We simulate the entire system by applying the Floquet (*i.e.*, periodic) boundary condition at the sides of the unit cell that composes the GT metasurface. The metasurface is designed as a reflective surface illuminated by a plane wave normally incident from above, with right- and left-circular polarizations, in the spectral range of 0.4–1.4 μm. The bottom face of the Al reflector layer is modeled as a Perfect Electrical Conductor (PEC). The reflectance response of the metasurface can be modulated by applying an external DC magnetic field along the *z*-direction, which influences the ferromagnetic properties of the Co meta-atoms and Co coating layer (see Figure 3(c)) and Figure 11S in Supplementary Information), by generating the off-diagonal terms in the dielectric response of the Co elements (see Eq. (1)). The values of the *ε*_*xy*_ and *ε*_*yx*_ terms used in the simulations correspond to an external magnetic field of 0.67 T [17].

The key physical processes involved in this system are as follows:

### 2.1 The localized surface plasmon resonance (LSPR)

Each meta-atom consists of a Co disk-shaped nanostructure, in which the free conduction electrons collectively oscillate in response to incident electromagnetic radiation. This collective electron oscillation, known as LSPR, couples strongly with the local electromagnetic field, leading to enhanced near-field intensities around the nanostructure. The resonance frequency of this LSPR mode depends sensitively on several factors: the geometric parameters of the meta-atom (including its size and shape), the intrinsic dielectric function of Co (which dictates how its free electrons respond to electromagnetic excitation), and the optical properties of the surrounding environment. In particular, the refractive index of the SiO_2_ layer influences the effective dielectric environment, thereby shifting the resonance frequency and modifying the strength of the plasmonic response. For a detailed analysis, refer to Figure 6S in the Supplementary Information. Figure 6S displays the extinction, absorption, and scattering cross sections of an isolate Co nanodisk, both in air and on a SiO_2_ substrate, across a range of wavelengths. These spectra highlight the spectral position and intensity of the LSPR. Additionally, the corresponding electric field distributions are shown, illustrating the spatial localization and enhancement of the electromagnetic field near the nanodisk.

### 2.2 The resonances of the GT cavity modes

In our system, the Co meta-atoms serve as a semi-reflective layer, while the bulk Al substrate acts as a totally reflective layer; taken together, they constitute a GT resonator, whose optical length (and thus its allowed cavity modes) is mainly determined by the thickness of the SiO_2_ dielectric spacer. The thin layer of Co along with the Co meta-atoms allows for the modulation of these cavity modes in the presence of an external magnetic field and circularly polarized light (see Figure 3(c)).

The wavelengths corresponding to the reflectance maxima arising from cavity modes within the dielectric spacer can be determined using the following equation [34]:

(2) $$\lambda_{\max}=\frac{2n_{\text{eff}}t_{d}}{m}$$

where *n*_eff_ denotes the effective refractive index, which depends on both the refractive indices of the materials comprising the cavity and the geometrical factors of the cavity; *t*_*d*_ is the thickness of the dielectric spacer, and *m* is the order of the considered cavity mode.

Conversely, the wavelengths corresponding to the reflectance minima due to cavity modes are given by the formula [34]:

(3) $$\lambda_{\min}=\frac{2n_{\text{eff}}t_{d}}{m+\frac{1}{2}}$$

### 2.3 The surface lattice resonances (SLRs)

**Figure 2:**
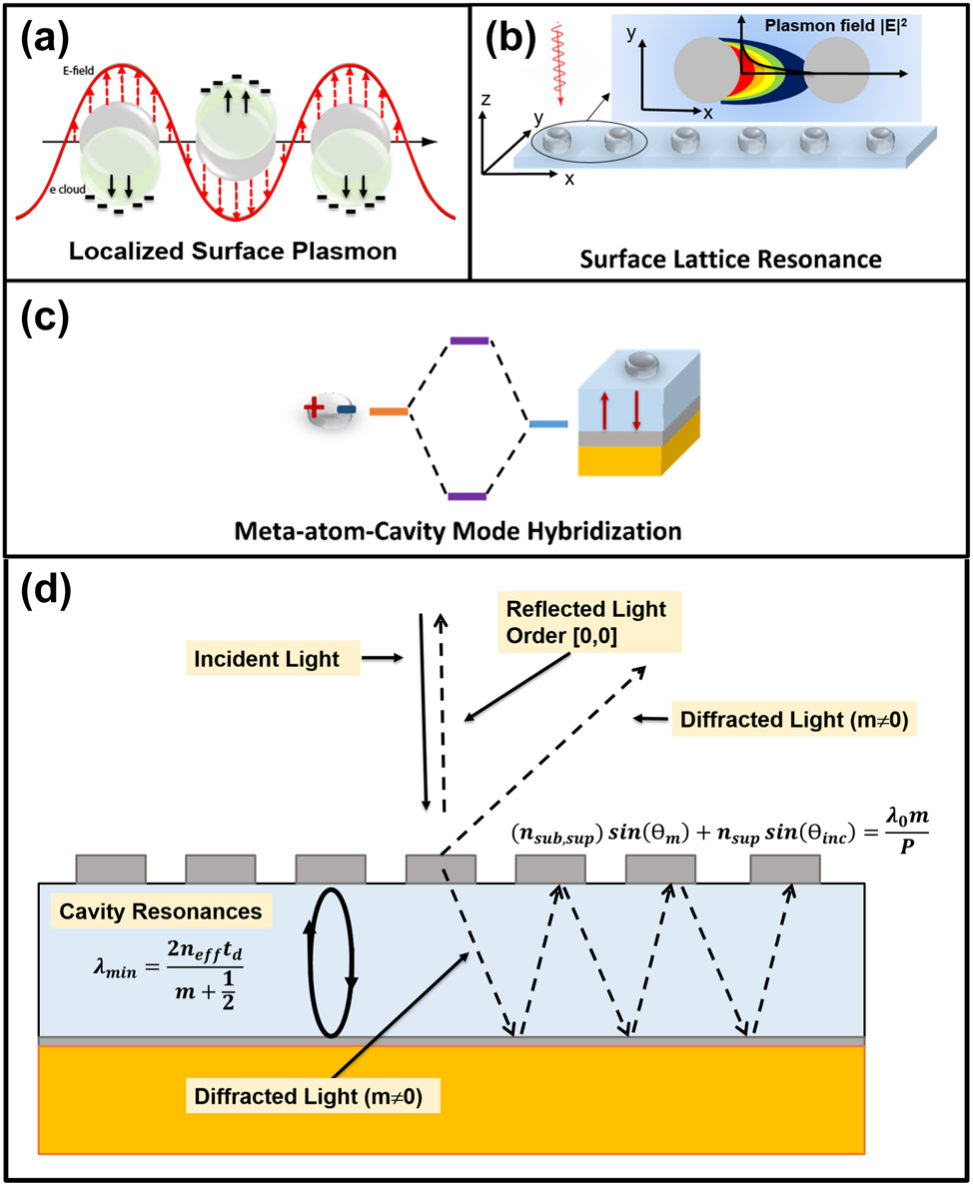
Optical phenomena involved in our metasurfaces. Schematic representation of localized surface plasmon resonance (a), surface lattice resonance (b), and meta-atom-cavity mode hybridization (c). The plus and minus signs visually represent the distribution of oscillating charges. (d) Schematic representation of the main optical phenomena associated with the grating and the cavity, excluding plasmonic resonances. See Equation (5) for the definitions of *n*_sub_, *n*_sup_, *m*, *θ*_*m*_, and *θ*_inc_.

SLRs [35], [36] occur when the wavelength of the incident light satisfies the condition for exciting a grazing diffraction mode that matches a diffracted order in a periodic nanostructure. In the present case, this condition can be verified for both the standard diffraction orders of the grating (either in air or inside the dielectric), and by the so-called Wood anomalies [36], that arise when the plasmonic resonance [37] wavelength of the structures composing the grating (*i.e.*, the disks) matches the grating period. The phase-matching condition for the excitation of SLRs in normal incidence can be expressed as:

(4) $$P=\frac{m\lambda_{0}}{n}$$

where *P* is the periodicity of the lattice, *λ*_0_ is the wavelength in the vacuum. *n* is the index of refraction of air or of the substrate, for grating grazing mode; in the case of Wood anomalies, *n* is given by the expression $n={\left[\left(\varepsilon_{\text{sub}}\varepsilon_{\text{met}}\right)/\left(\varepsilon_{\text{sub}}+\varepsilon_{\text{met}}\right)\right]}^{\frac{1}{2}}$ where *ɛ*_sub_, *ɛ*_met_ are the real parts of the dielectric permittivity of the substrate and of the metal, respectively. Satisfying this condition enables the coupling of incident light to graze surface waves, which in turn mediates coherent optical interactions between adjacent meta-atoms (see Figure 2(b)).

### 2.4 Planar dielectric/metallic waveguide and diffraction grating

The SiO_2_ spacer/Al reflector assembly acts as a planar dielectric/metallic waveguide, which can support propagating modes parallel to the dielectric plane [38]. Besides, the array of Co disks constitutes a diffraction grating. The propagating modes in the waveguide are excited by the diffraction of the incident electromagnetic wave on the grating structure, transmitted into the dielectric. Moreover, the grating originates also diffracted waves in the overlying air. The direction of the diffracted beams in air and in the dielectric are governed by the general grating equation:

(5) $$\left({\text{n}}_{\text{sub},\sup}\right)\sin\left(\theta_{m}\right)+{\text{n}}_{\sup}\sin\left(\theta_{\text{inc}}\right)=\frac{\lambda_{0}m}{P}$$

In this expression, *m* is an integer representing the diffraction order. *n*_sub_ and *n*_sup_ denote the refractive indexes of the upper and lower dielectric, *θ*_inc_ and *θ*_*m*_ correspond to the propagation angle of the incident and of the diffracted beam. In the first addend, we need to use *n*_sup_ if the output wave is diffracted in the half space of the incoming beam, or *n*_sub_ if it is diffracted into the lower dielectric space.

All these processes are exemplified in Figure 2.

**Figure 3:**
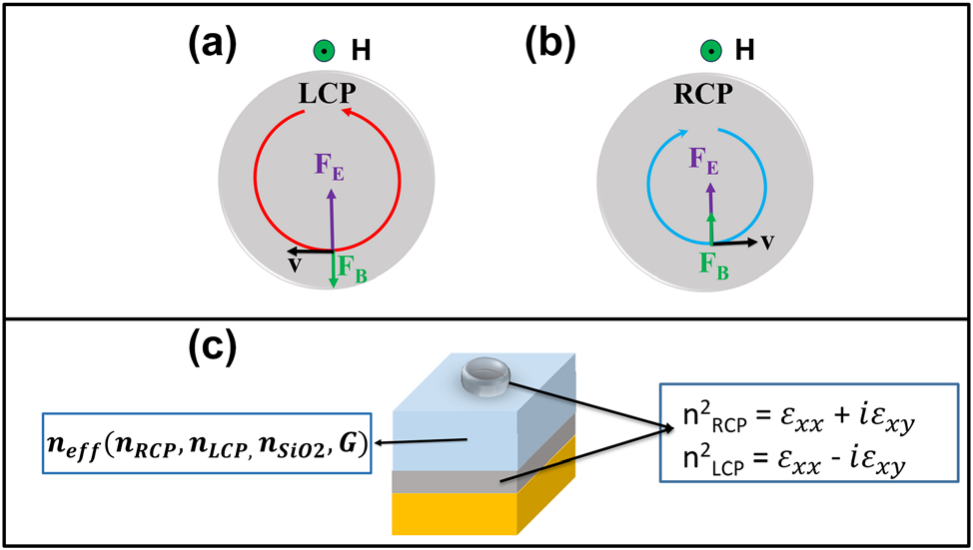
Principles of magneto-optics. (a) and (b) Illustration of the effects of the magnetic force **F**_**B**_ = *q*(**v** × **B**) with **B** = *μ*_0_(**H** + **M**) and the electric force **F**_**E**_ = *q* **E** (together constituting the Lorentz force) on an electron with velocity *v* associated with the oscillating electron density on the meta-atom surface in the presence of LCP and RCP light. (c) A Gires–Tournois cavity is used to enable multiple interactions between the Co elements and the incident light, thereby enhancing the magneto-optical Kerr effect (MOKE). The cavity also allows modulation of the MOKE response by altering boundary conditions and the effective refractive index, which depends on the cavity’s geometric factor (**G**) and the refractive indices of the constituent materials.

The external magnetic field (**H**) modifies the permittivity of Co by introducing off-diagonal terms in its dielectric tensor affecting both the base layer and the plasmonic meta-atoms [14], [39], [40], [41], [42]. The off-diagonal terms are related to the Lorentz force acting on free charges participating to LSPR (Figure 3), which is due to both the external magnetic field and the internal magnetization (**M**) of the material. This process also alters the cavity modes, as illustrated in Figure 11S of the Supplementary Information, and the surface lattice modes, as previously reported in Ni nanodisks periodic lattices [43]. In particular, resonant cavity modes enable multiple reflections of light on the magneto-optical Co nanostructures and Co layer, significantly enhancing the *p*-MOKE. Furthermore, the coupling of LSPRs and SLRs with GT cavity resonances enables strong spatial confinement of the electromagnetic field within the Co structures. This results in a higher local field intensity where the magneto-optical activity originates, thereby amplifying the *p*-MOKE response. These couplings between GT, LSPRs, and SLRs result in a complex shape of the reflectance spectra characterized by significant modulations and strong variations in both reflectance and phase delay across different wavelengths. This behavior is much more complex with respect to that observed with isolated ferromagnetic nanostructures and multilayered system without the Co nanostructures on top (see Supplementary Information: Figures 7S–10S).

## 3 Results and discussion

In order to comprehend the *p*-MOKE and the general response of ferromagnetic systems, we systematically analyzed the differential effects on the reflectance of RCP and LCP light under an external magnetic field. Indeed, linearly polarized light (typically used in MOKE spectroscopy) can be seen as the sum of RCP and LCP light. Consequently, MOKE ellipticity and rotation can be also described in terms of differential phase and absorption/reflection intensity of the two circular polarizations. It must be noticed that reversing the direction of the magnetic field is equivalent to inverting the handedness of the incoming electromagnetic wave (e.g., passing from LCP to RCP).

We conducted a comprehensive optimization of the Co disk dimensions and spacing, along with the thickness of the SiO_2_, and Co layers to maximize the phase and intensity variation of the reflected waves when the metasurface interacts with RCP and LCP light under the influence of an external magnetic field.

To accurately characterize the magneto-optical response of our simulated systems, we begin our analysis by considering a representative GT metasurface consisting of a bulk Al bottom layer, a 0.015 μm thick Co ferromagnetic layer, a 0.500 μm thick SiO_2_ layer, and a 0.015 μm thick Co disk with a radius (*R*) of 0.120 μm positioned on top of the SiO_2_ layer (see Figure 1). The periodicity (*P*) of the unit cell is 0.500 μm.

The most important output of our simulations are the complex reflectance spectra for the electric field amplitude for LCP and RCP, *i.e.*, $r_{\text{LCP}}\left(\lambda \right)$ and $r_{\text{RCP}}\left(\lambda \right)$ . From these, we calculate the corresponding reflectances for the intensities, *i.e*., $R_{\text{LCP,RCP}}\left(\lambda \right)={\left|r_{\text{LCP,RCP}}\left(\lambda \right)\right|}^{2}$ , and their phases, *i.e*., $\varphi_{\text{LCP,RCP}}\left(\lambda \right)=\arg\left(r_{\text{LCP,RCP}}\left(\lambda \right)\right)$ (see also Equations (3) and (5)–(8) in Supporting Information).

**Figure 4:**
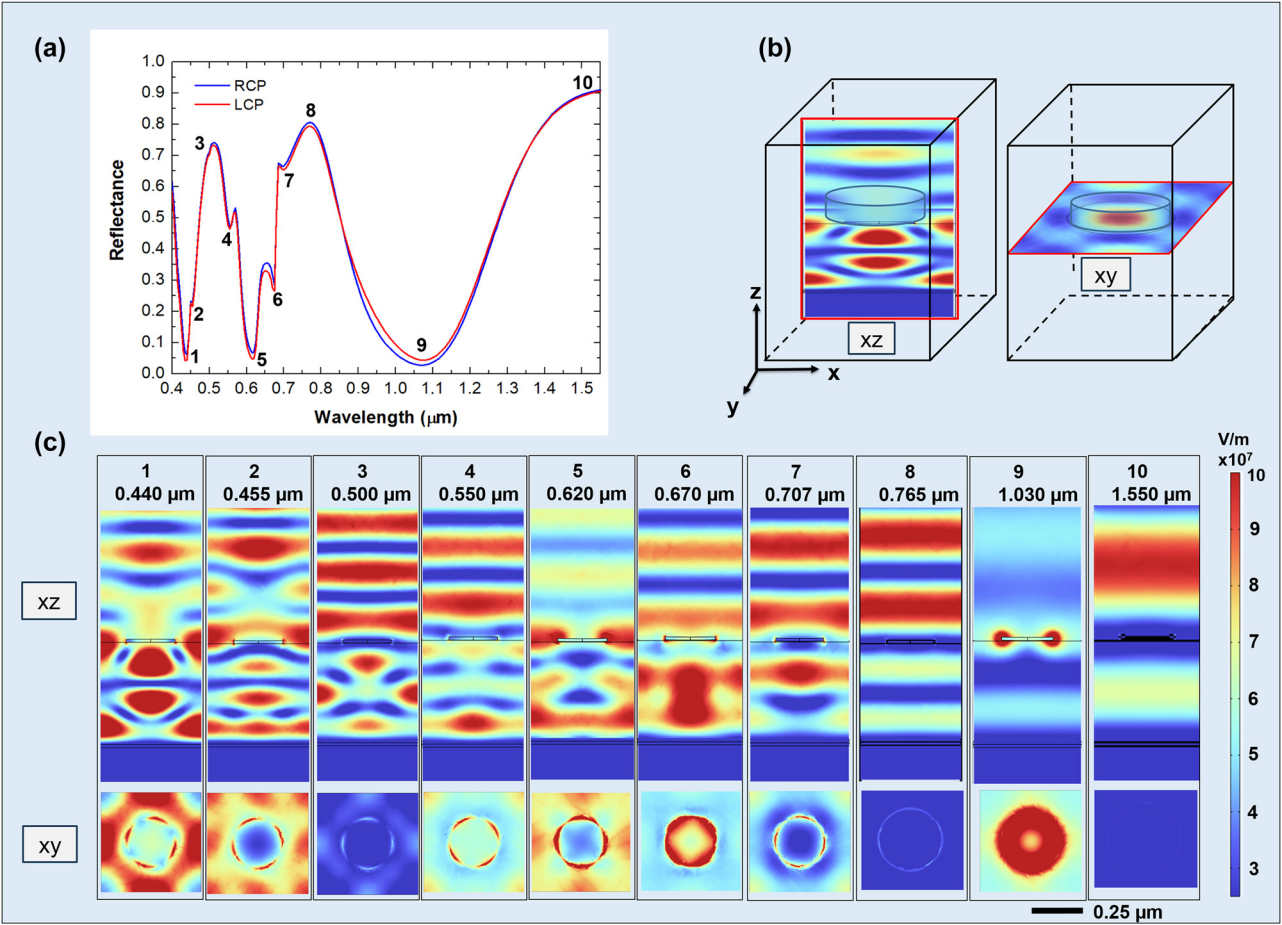
Analysis of resonant modes. Reflectance spectra for RCP (blue) and LCP (red) light (a), the position of the *xz* and *xy* plane with respect to the meta-atom structure (b), and the corresponding electric field intensity distributions for RCP light at the reflectance minima (c). The *xz* view shows the electric field amplitude distribution across a vertical plane passing through a meta-atom diameter; the *xy* view illustrates the intensity distribution on a plane in proximity to the disk-shaped meta-atom. All the graphs share the same color scale for the values of the electric field.

Figure 4(a)–(c) displays the calculated intensity reflectance spectra $R_{\text{LCP,RCP}}\left(\lambda \right)$ for RCP and LCP light (**a**), as well as the local electric field intensity distributions at the reflectance minima (**b**) for the metasurface with *P* = 0.500 μm.

To analyze the complex behavior depicted in Figure 4, we start analyzing the reflectance spectrum of Figure 4(a). The reflectance minima at wavelengths of 1.030 μm, 0.620 μm, and 0.440 μm (states labeled with 1, 5, and 9 in Figure 4(a)) are partially due to GT cavity modes as predicted by Equation (3). The peaks related to the states 3, 8, and 10 mainly correspond to the GT reflectance maxima (Equation (2)). The GT cavity modes were calculated using Equations (2) and (3) with a refined effective refractive index (*n*_eff_) of 1.54 (see also Figure 10S in Supplementary Information). This simple behavior is nonetheless complicated by the mutual interaction between the GT modes, the LSPRs, and the SLRs, as we are going to discuss below.

To further elucidate the reflectance spectrum behavior, it is instructive to analyze the states pictured in Figure 4(c) sequentially from longer to shorter wavelengths, *i.e.*, from state 10 to state 1. The geometry of the *XY* and *XZ* cutoff planes used to analyze the field distribution is depicted in Figure 4(b).

At 1.55 μm, the metasurface behaves as a simple GT cavity in reflection, with a significant coupling of the incident radiation into the dielectric layer, as shown in the picture relative to the state 10. The *XY* distribution is quite uniform in both *x* and *y* direction, with no indication of field enhancement around the Co nanodisks. This confirms the dominance of a dielectric GT-like mode, where the meta-atoms are seen by the electromagnetic wave as a continuous thin conductive layer.

For decreasing wavelengths, the LSPR resonance of the individual disks gets progressively excited, up to 1.030 μm (see also Figure 6S in Supporting Information where the LSPR of isolated Co nanodisk on SiO_2_ is shown), determining a broad and deep minimum in the reflectance (state 9), due to the dissipation in the plasmon. This is associated with a significant enhancement of the electric field amplitude in correspondence of the disks, whose amplitude distribution in the proximity of the disk has cylindrical symmetry around the *z* axis. The corresponding *XY* slice shows an intense, highly symmetric circular profile centered on each disk, characteristic of a strong dipolar LSPR mode. Additionally, the field distribution along the periodic direction of the metasurface reveals partial spatial overlap between the near fields of adjacent meta-atoms, indicating an inter-disk coupling. This minimum in reflectance at 1.030 μm also coincides with a predicted dip in the reflectance of the GT cavity, as described by Equation (3), suggesting a hybridization between the LSPR and the cavity mode at this wavelength.

At 0.765 μm (state 8), we can observe the peak in reflectance due to the second order of the GT cavity (see Equation (2)), with only a small involvement in the dipolar resonance of the nanodisks. The *XY* field distribution remains relatively uniform, again indicating weak plasmonic involvement.

Further decreasing the wavelength, the grating structure comes into play. At 0.707 μm (state 7), the electric field is confined between the bottom surface of the disks and the dielectric substrate. In this case, the diffraction from the grating structure constituted by the Co disks excites propagating modes of higher order in the dielectric spacer, bounded between the bottom reflector and the SiO_2_–air interface. As the exciting radiation arrives at normal incidence, modes propagating in the positive *x* (*y*) and negative *x* (*y*) directions are both equally excited in the dielectric, resulting in the formation of a standing wave pattern. Surface lattice resonance of the grating with period $P=\sqrt{2}\times 0.500\;\;\mu m$ (see also Figure 24S in Supporting Information), corresponding to coupling between disks along the diagonals (Equation (4)), can be also seen: indeed the *XY*-plane electric field map exhibits periodic features with symmetry along the diagonal of the square unit cell. At 0.670 μm (state 6), we observe a larger involvement of the LSPR (see also Figure 6S in Supporting Information), as evidenced by the higher value of the electric field near the lower edges of the Co disk. This is reflected in electric field distribution in the *XY* plane with the formation of more localized hot spots at the disk perimeter.

In state 5 (0.620 μm), coupling occurs between the LSPR of the individual Co nanodisks in the array through the excitation of SLR and the consequent occurrence of surface wave anomaly [36], [44] (see Figure 25S in Supporting Information). This surface lattice resonance is predicted by Equation (4), by inserting $n={\left(\frac{\varepsilon_{\text{Co}}\varepsilon_{\text{Si}{\text{O}}_{2}}}{\varepsilon_{\text{Co}}+\varepsilon_{\text{Si}{\text{O}}_{2}}}\right)}^{\frac{1}{2}}$ . Indeed, focusing solely on the portion of the electric field distribution on the disk, it is evident that in state 5, the electric field is confined at the interface SiO_2_/air in the region between the disks, highlighting strong coupling between the LSPR of adjacent disks. In the *XY* plane, an extended pattern connecting neighboring disks emerges, indicating near-field coupling. In state 4 (0.550 μm), we observe again coupling between disks along the diagonals and an electric field intensity concentrated within the dielectric spacer due to the build-up of propagating modes in the dielectric layer excited by the grating diffraction. We also note a weak contribution from the Co disk’s LSPR, evidenced by field concentration at the edges of the meta-atoms.

At 0.500 μm (state 3), the wavelength in air matches the periodicity of the structure, so that a diffracted surface wave propagating at the interface between the dielectric and the air is excited. This couples the response of individual nanodisks, allowing excitation of SLR, which are only partially coupled with other phenomena (see *XY* slice). Simultaneously, at this same wavelength, a GT resonance is also excited as predicted by Equation (2).

In state 2 (0.455 μm), the observed electric field distribution results from grating diffraction occurring both within the dielectric spacer and in air, accompanied by significant electric field enhancement near the edges of the nanodisks due to LSPR.

Finally, in state 1 (0.440 μm), hybridization occurs between a LSPR mode of the isolated Co nanodisk and the GT cavity mode (see Equation (3)). This is evident from the very high electric field concentration inside the dielectric (see Figure 4(c), *xz* view), together with a more attenuated, but still non-negligible electric field concentration near the edges of the Co disk. Notably, in state 1, the electric field is more intensely confined within the dielectric compared to state 2. The *XY* slices show strongly localized, 4-fold symmetric hot spots at the disk rims, particularly intense in state 1. Furthermore, the grating formed by the nanodisks array diffracts part of the incoming wave into both the dielectric substrate and the surrounding air.

Stronger effects of the magnetic field on the reflectance spectrum are observed when the GT cavity modes are hybridized with LSPR, as indicated by the increased reflectance contrast between RCP and LCP polarizations, particularly for states 1 (0.440 μm), 5 (0.620 μm), 6 (state 6), and 9 (1.030), see Figure 6(a).

**Figure 5:**
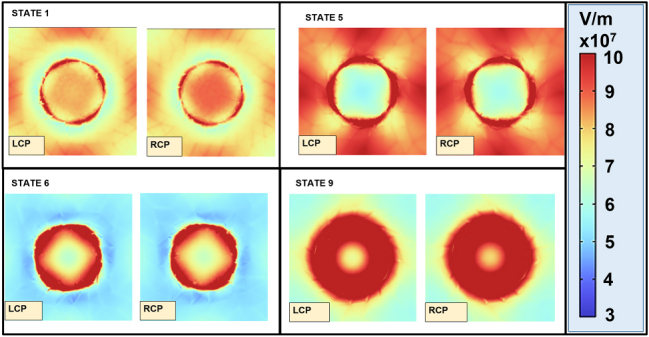
Magneto-optical electric field response. Effects of the external magnetic field on the electric field distribution in the presence of LCP and RCP light for states 1, 5, 6, and 9.

To further elucidate this effect, Figure 5 shows the electric field distributions for states 1, 5, 6, and 9 in the *xy*-plane (at the interface between SiO_2_ and disk-shaped meta-atom), comparing the metasurface response to RCP and LCP polarizations under an external magnetic field of 0.67 T. It can be seen that for the states 1, 5, and 6, the field amplitude distribution at the edges of the disk has a 4-fold rotational symmetry. In states 1 and 5, the regions with highest field amplitude of the disk approximatively face the nearest neighbors along the *x* and *y* direction, indicating a strong coupling between the disks in these directions; the slight pattern rotation, either counter-clockwise or clockwise for LCP and RCP, respectively, results from the interaction of carriers with the magnetic field. In state 1, there is also an appreciable increase of the field amplitude in the central region of the disk for RCP, in comparison with LCP (see Figure 3(a) and (b) and Figure 2S in Supporting Information). In state 6, the high amplitude regions are aligned toward the neighbor disks in the diagonal directions, again with a slight rotation either counter-clockwise or clockwise for LCP and RCP, respectively; this suggests a coupling between diagonal elements, perturbed by the magneto-optical effect. In the case of state 9, the field distribution features complete rotational symmetry, indicating that the coupling with neighbors is weak; again we can see that the field amplitude in the central region of the disk is higher for RCP than for LCP as in the case of state 1, see Figure 3(a) and (b) and Figure 2S in Supporting Information.

After illustrating and describing the main magneto-optical phenomena involved in these systems, we proceeded to explore the space of geometrical parameters. In particular, we investigated the magneto-optical behavior of these GT metasurfaces by varying the periodicity (*P*) of the unit cell, starting from larger values (*P* = 1.00 μm) and reducing to smaller values (down to *P* = 0.280 μm), while keeping all other parameters fixed. At *P* = 0.280 μm, the distance between each disk is just 0.020 μm, approaching the limit where the Co layer would become continuous (filling factor of 1). However, by dealing with meta-atoms rather than with a continuous layer, we can still modify the spectral reflectance shape by adjusting the shape of the meta-atoms. To illustrate the importance of meta-atom shape and provide a comparison with the metasurface at *P* = 0.500 μm, Figures 13S and 14S in the Supplementary Information show the reflectance spectra for two metasurfaces with the same value of *P* = 0.280 μm, but featuring either disk-shaped or square-shaped meta-atoms, respectively. In both cases (and in particular for the square-shaped meta-atoms), the reflectance spectra are simpler, with three dips corresponding predominantly to the hybridization between GT cavity modes and the LSPR of individual meta-atoms. Although the close spacing between meta-atoms leads to significant near-field overlap of their localized plasmon fields, coupling between SLRs and LSPRs occurs only at 0.396 μm (Figure 24S in Supporting Information) and 0.420 μm (see Figure 13S) within the investigated spectral range. This is because the lattice periodicity does not satisfy the condition necessary to support grazing-angle diffracted modes at the interface, which are a prerequisite for SLR formation. In order to obtain a better understanding of the variation of the complex reflectance of the metasurface for LCP and RCP light in presence of an external magnetic field, we calculated also the reflectance contrast spectrum (expressed as percentage) and the phase difference spectrum (see Equations (6) and (7)).

(6) $$\frac{\Delta R}{R}\text{\% }=\left(\frac{R_{\text{RCP}}\left(\lambda \right)-R_{\text{LCP}}\left(\lambda \right)}{\frac{R_{\text{RCP}}\left(\lambda \right)+R_{\text{LCP}}\left(\lambda \right)}{2}}\right)\cdot 100$$

(7) $$\Delta \phi =\varphi_{\text{LCP}}\left(\lambda \right)-\varphi_{\text{RCP}}\left(\lambda \right)$$

**Figure 6:**
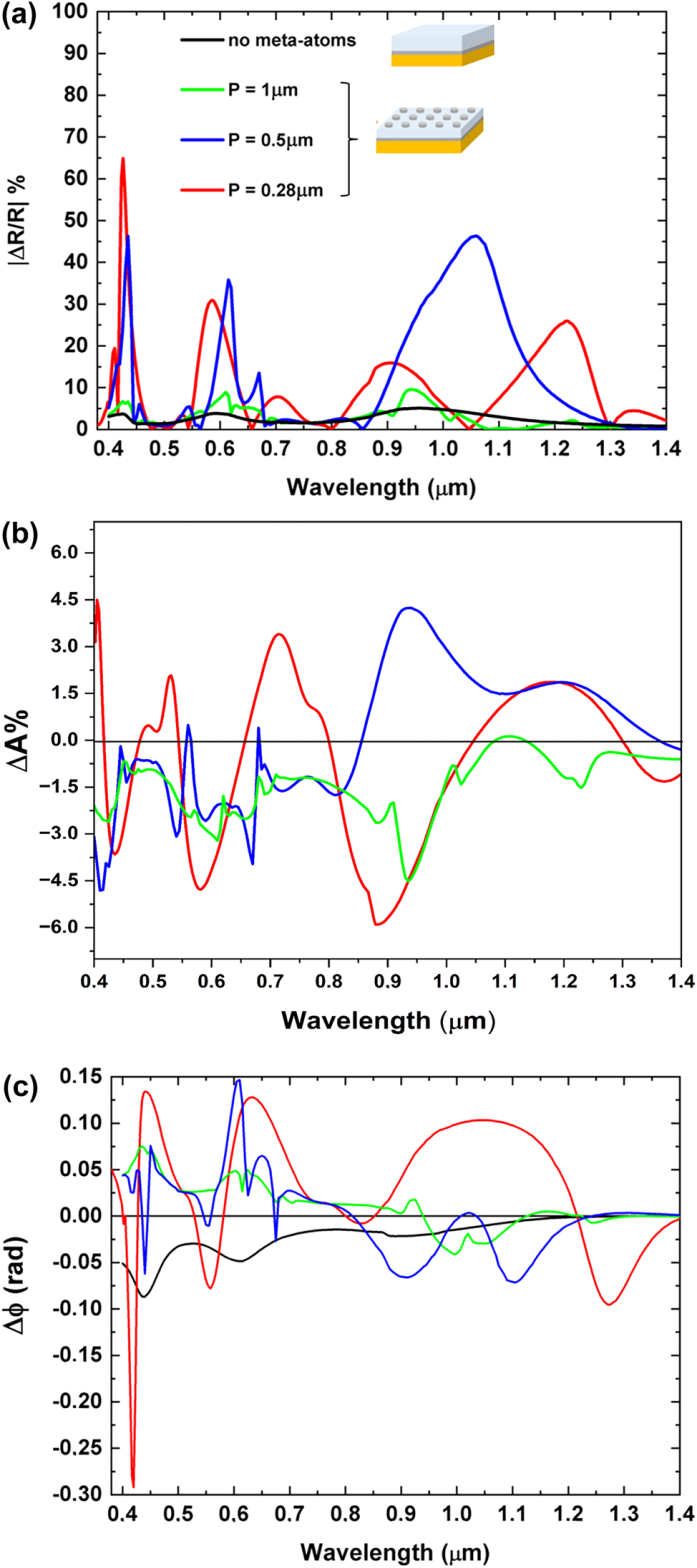
Spectral analysis of reflectance contrast, MCD, and phase delay. (a) Absolute values of percentage reflectance contrast between RCP and LCP (Δ*R* = *R*_RCP_ − *R*_LCP_) polarizations for cells with different periods, *P*. (b) MCD (Δ*A*% = (*A*_RCP_ − *A*_LCP_)100) for the same GT metasurfaces. (c) Summary of the phase delay variation (Δ*ϕ* = *ϕ*_RCP_ − *ϕ*_LCP_) observed in GT metasurfaces composed of disk-shaped meta-atoms at various cell periods. Black line: response of GT resonator without meta-atoms; green line: GT metasurface with a period *P* = 1.00 µm; blue line: GT metasurface with *P* = 0.500 µm; red line: GT metasurface with *P* = 0.280 µm.

The spectra of reflectance difference, Magnetic Circular Dichroism (MCD), and phase difference were calculated for different geometrical arrangements, in particular by changing *P* while maintaining fixed the other parameters. The MCD was evaluated as Δ*A* = *A*_RCP _− *A*_LCP_ = (1 − *R*_RCP_) − (1 − *R*_LCP_) = *R*_LCP_ − *R*_RCP_. These results are shown in Figure 6(a)–(c).

The analysis begins with *P* = 1.00 µm, then *P* is halved to *P* = 0.500 µm, and finally set to *P* = 0.280 µm (slightly more than half of 0.500 µm), to progressively reduce the disk spacing, but still maintaining isolated disks.

As illustrated in Figure 6(a)–(c), the increase of the Co nanodisks’ volume fraction (achieved by reducing the unit cell period and thus overall increasing the packing density of the ferromagnetic meta-atoms) does not translate into a straightforward enhancement of the reflectance contrast under an external magnetic field. Notably, while decreasing the metasurface period effectively raises the density of ferromagnetic meta-atoms, certain spectral regions exhibit higher reflectance contrast at an intermediate period (e.g., *P* = 0.500 μm) compared to the reflectance contrast observed at a smaller period (*P* = 0.280 μm), where the meta-atom density is greater. This non-monotonic behavior underscores the pivotal role played by the coupling between different optical modes (GT cavity modes, grating diffraction, LSPR, and SLR) in governing the magneto-optical response of the system. Such interactions facilitate precise tuning of the magneto-optical properties through careful adjustment of structural parameters, such as the unit cell period or meta-atoms shape and dimensions. This behavior is further elucidated in Supporting Information (Figures 15S–23S), which illustrates the impact of various structural parameters on the magneto-optical response of the metasurface at a fixed wavelength of 0.900 µm. More specifically, at wavelengths of 0.620 µm, 0.670 µm, and 1.030 µm, the metasurface with a period of *P* = 0.500 μm exhibits a pronounced reflectance contrast, which surpasses that of the metasurface with the smaller period (*P* = 0.280 μm). This enhanced response arises from the simultaneous presence and hybridization of multiple optical phenomena at these wavelengths, *i.e.*, LSPR, SLR, grating diffraction, and GT cavity modes.

The interplay and coupling among resonant modes enable the strategic design of magneto-optical and spectral properties, allowing enhanced magneto-optical responses to be achieved even in metasurfaces with lower meta-atom densities (*i.e*., larger unit cell periods). While the density of meta-atoms is indeed an important parameter influencing the optical and magneto-optical behavior, it is not the primary driver of performance enhancement. As demonstrated in our results, the coupling between different optical modes plays a central role in achieving high performance, even in less densely packed arrays of magneto-optical materials.

This interplay is clearly observed in Figure 6(a)–(c), where the metasurface with a reduced period of *P* = 0.280 μm exhibits a strong reflectance contrast of approximately 65 % and a notable phase delay of about 0.3 radians (≈14°) at a wavelength of 0.420 µm. While the higher density of meta-atoms contributes to stronger overlap of local plasmonic fields, this enhancement cannot be solely attributed to geometric packing.

Figure 13S in the Supporting Information further support this, showing that the electric field distribution at 0.420 µm reveals an in-plane, long-range interaction mediated by the field inside the air–dielectric interface, with field vectors extending across neighboring unit cells. This spatial pattern indicates strong hybridization between the LSPR of individual nanodisks and the SLR that emerges along the diagonal of the square lattice with $P=\sqrt{2}\times 0.280\;\;\mu m$ , on the lower side of the Co disk–dielectric interface.

This Wood anomaly is predicted by Equation (4), by inserting $n={\left(\frac{\varepsilon_{\text{Co}}\varepsilon_{\text{Si}{\text{O}}_{2}}}{\varepsilon_{\text{Co}}+\varepsilon_{\text{Si}{\text{O}}_{2}}}\right)}^{\frac{1}{2}}$ and $P=\sqrt{2}\times 0.280\;\;\mu m$ .

The presence of a GT cavity mode at this wavelength further amplifies this effect, enhancing both the amplitude and phase variation of the metasurface for LCP and RCP.

This finding has interesting implications for the design of future optical systems that are magnetically tunable.

**Figure 7:**
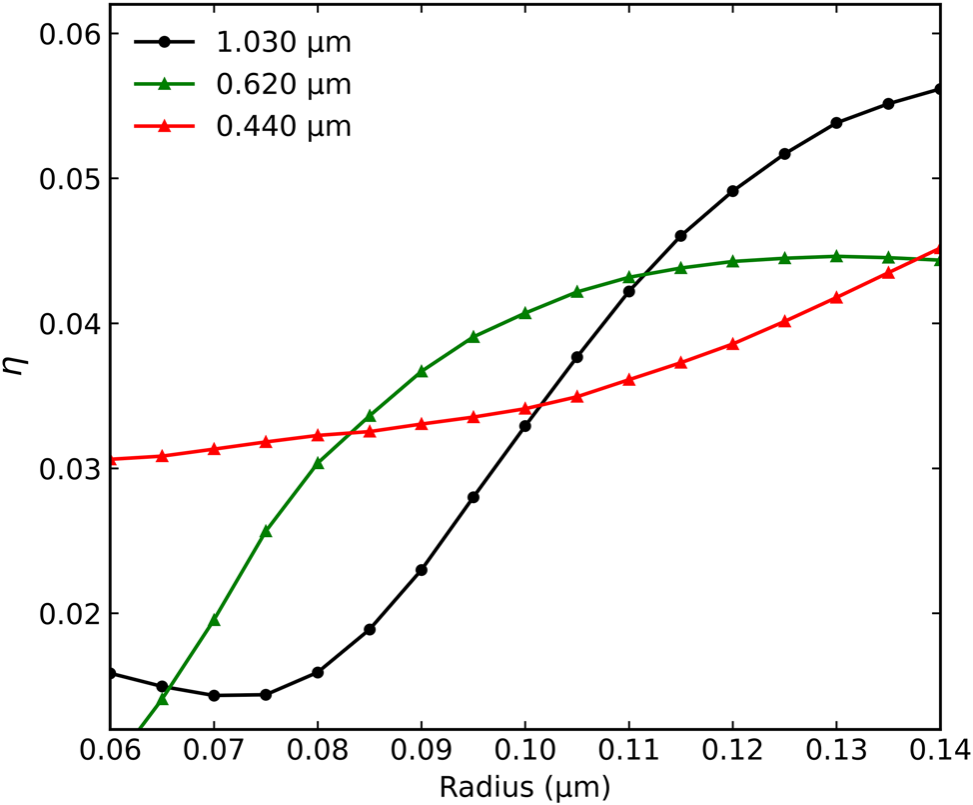
Geometric optimization of magneto-optical response. FoM (*η* = |*r*_RCP_ − *r*_LCP_|) as a function of disk radius for the metasurface with *P* = 0.500 μm (and other parameters from Figure 4), calculated at the wavelengths of states 1 (0.440 μm), 5 (0.620 μm), and 9 (1.030 μm).

To better understand the selection of the meta-atom dimensions, we analyzed the influence of the disk radius on the overall magneto-optical response. Figure 7 shows this dependence at the key wavelengths of the GT cavity modes identified in Figure 10S. We quantify the total magneto-optical effect using the Figure of Merit (FoM), defined as *η* = |*r*_RCP_ − *r*_LCP_|, where *r* is the complex amplitude reflectance. This value *η* directly measures the total difference in the optical response (both amplitude and phase) between RCP and LCP light.

At 440 nm (red line), which corresponds to a small hybridization of LSPR and a GT mode (state 1), the response *η* shows a relatively low dynamic and a quasi-linear dependence on the radius. This suggests that while coupling is present, the strong radius-dependence of the LSPR is not the dominant factor, but the diffraction phenomena that occurs, which are dependent to periodicity of the disks grating.

In contrast, at 1,030 nm (black line, state 9, GT + LSPR) and 620 nm (green line, state 5, SLR + GT + LSPR), *η* shows a much larger dynamic and a strong nonlinear dependence on the radius. The response increases significantly as the radius grows from 0.08 μm to 0.12 μm, where the curves begin to peak or saturate. This analysis confirms that the chosen radius of *R* = 0.120 μm places the metasurface in a region of optimized geometric parameters, where the synergistic coupling between plasmonic, cavity, and lattice modes is highly sensitive to an external magnetic field and results in a strong overall magneto-optical response.

These results are reported in Figure 7.

**Figure 8:**
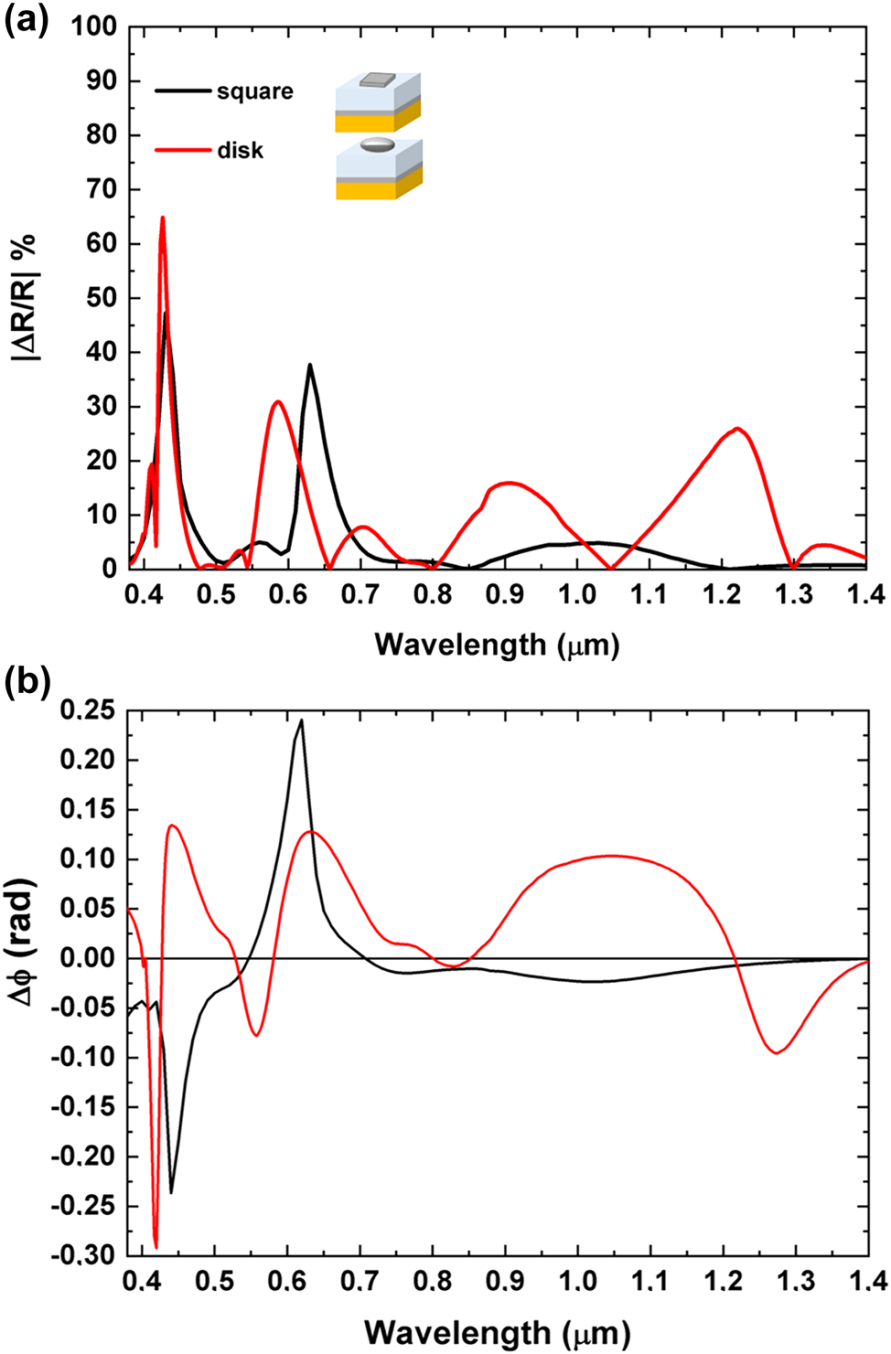
Spectral analysis of reflectance contrast, and phase delay. (a) Absolute values of percentage reflectance contrast illustrating the reflectance differences between RCP and LCP polarizations for cells with *P* of 0.28 µm with square shaped meta-atoms (black line) or disk-shaped meta-atoms (red line). (b) A comparative analysis of metasurfaces phase variations for RCP and LCP lights featuring disk-shaped and square-shaped meta-atoms, both with a cell period P of 0.280 µm.

To demonstrate the significant influence of geometric parameters on the magneto-optical response of a GT metasurface, Figure 8 presents the percentage reflectance contrast (**a**) and phase delay variations (**b**) obtained by substituting disk-shaped meta-atoms with square-shaped meta-atoms in a GT metasurface with *P* = 0.280 μm.

This substitution shows how varying the shape of the meta-atoms can affect the magnetic modulation, thereby providing further opportunities to finely tune the optical properties of the metasurface.

We also tested meta-atoms with inherently birefringent geometries, replacing the original disks and squares, to observe their differential response to RCP and LCP light while applying an external magnetic field (*H*) to amplify these effects. As a simple case study, we chose ellipse-shaped meta-atoms.

**Figure 9:**
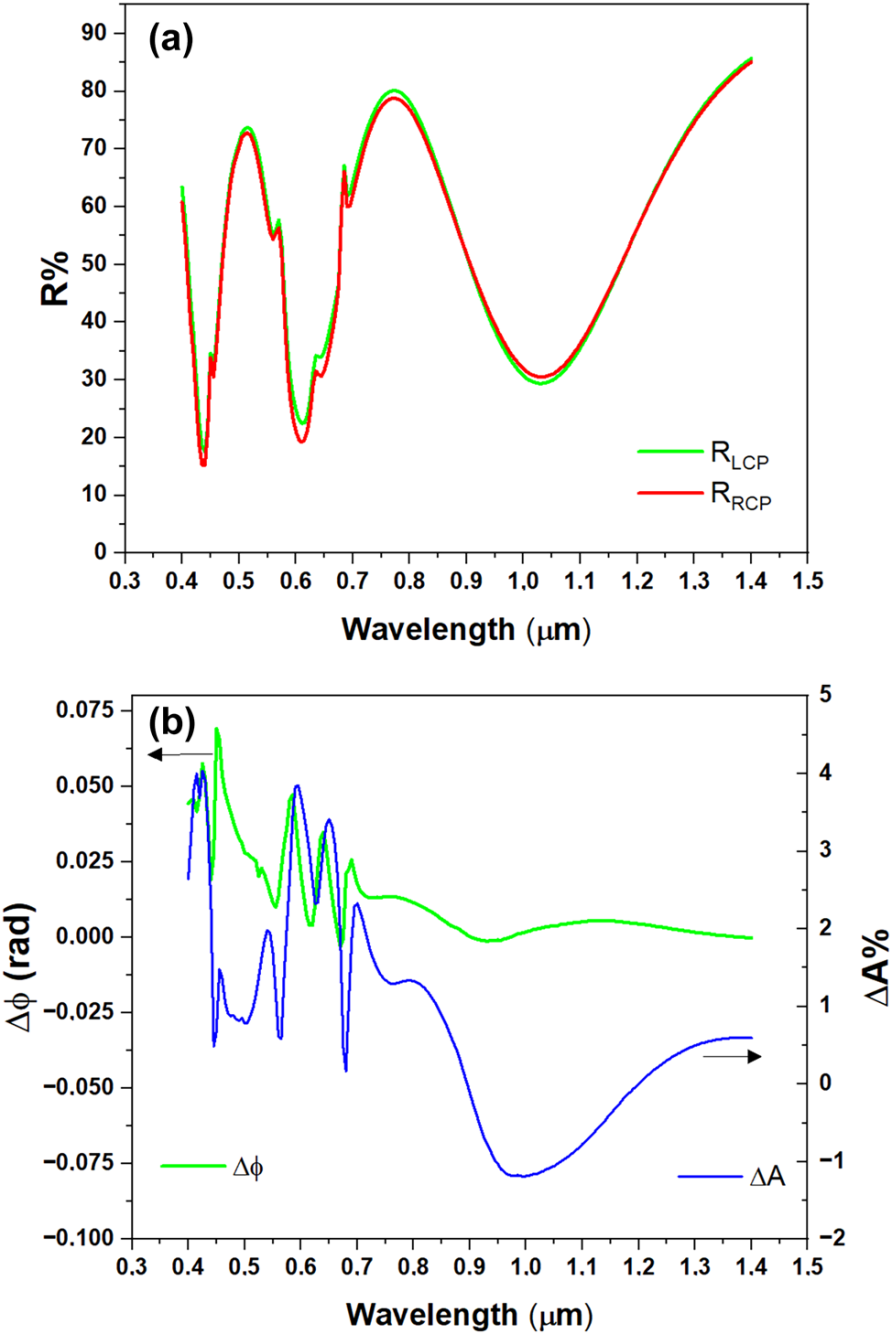
Reflectance, MCD and phase delay for ellipse shaped meta-atoms. (a) Reflectance spectra for RCP and LCP for a square pattern of ellipse shaped meta-atoms. All the other parameters as in the simulation of Figure (4); (b) phase difference between LCP and RCP, and MCD (absorption difference between RCP and LCP), for the metasurface with triangular pattern.

Figure 9 (a) and (b) reports reflectance spectra for this GT metasurface, along with the magnetic circular dichroism spectrum and the associated phase delay difference between LCP and RCP.

Overall, the data in Figure 8(a) and (b) show a similar trend for the analyzed physical quantities compared to the disk-shaped architecture (Figures 4 and 5). However, a key difference is clear in the reflectance spectrum: we observe shallower minima than in the case of disk-shaped meta-atoms. We attribute this to the weaker coupling between the GT cavity resonances and the plasmonic resonances.

This is a direct consequence of the new geometry. The selected nano-ellipses have the semi-axis *a* equal to the original disk radius (120 nm) along the *x* axis, but the semi-axis *b* of only half that value (60 nm) along the *y* axis. This demonstrates how intentionally modifying the plasmonic mode matching (by changing the meta-atom shape, size, and edge distance) provides direct control on the depth of the reflectance minima, as well as the resulting magnetic circular dichroism and phase delay between LCP and RCP.

**Figure 10:**
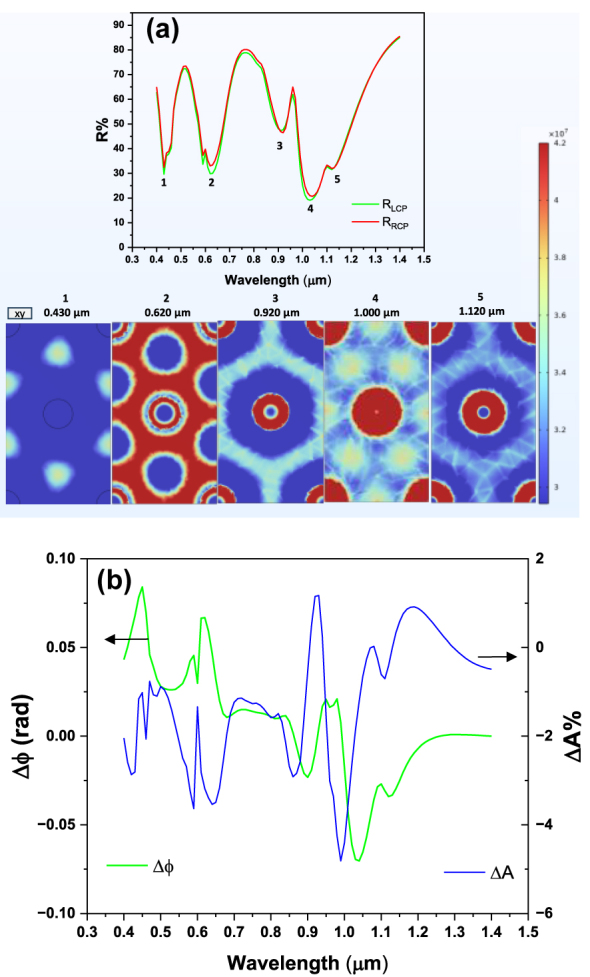
Reflectance, MCD and phase delay for triangular pattern of disks. (a) Reflectance spectra for RCP and LCP for a triangular disk pattern. Disk spacing is 0.5 μm, other parameters as in the simulation of Figure 4; (b) phase difference between LCP and RCP, and MCD (absorption difference between RCP and LCP), for the metasurface with triangular pattern.

In a separate design study, we employed an equilateral triangular lattice distribution for the Co meta-atoms, maintaining a primary center-to-center lattice constant (*P*) of 0.5 μm. All other design parameters (e.g., layer thicknesses, disk radius) were kept consistent with the detailed geometry presented in Figure 4. The corresponding simulated reflectance spectra for this triangular layout, along with the magnetic circular dichroism spectrum and the associated phase delay difference between LCP and RCP, are presented in Figure 10(a) and (b), respectively. As anticipated, the spectral characteristics deviate substantially from those of the square lattice configuration (Figure 4), confirming that the change in lattice symmetry introduces a modified set of allowed diffractive momentum matching conditions. This effect is most pronounced in the near-infrared region (1.0 μm), where the broad (LSPR-GT) hybrid resonance (which appeared as a single, broad feature in the square lattice) undergoes splitting into three distinct, closely spaced subpeaks, identified as states 3, 4, and 5. This is a direct consequence of the synergistic modal coupling, arising from the hybridization of the localized LSPR-GT mode with the grazing-incidence Surface Lattice Resonances (SLRs) of the triangular lattice. These specific SLRs are tied to the next-nearest-neighbor periodicity (*P*_eff_ ≈ 0.866 μm) and are determined to occur near 0.930 μm (*n*_eff_ = 1.06) and 1.100 μm (*n*_eff_ = 1.27), while in the square lattice, these modes are degenerate. This complex LSPR-GT-SLR triple hybridization is unique to this geometry and demonstrates the high degree of spectral tunability achievable by modifying the array symmetry. Crucially, despite the increased complexity of the coupled modes, the magneto-optical response remains robust: the MCD features exhibit peak values reaching a few percent, and the magnetically induced phase difference attains values approaching 80 mrad.

As shown in Figures 6(b), 9(b), and 10(b), the MCD values obtained for the GT-based structures are fairly high, and they reach notable magnitudes of +4.5 % and −6.1 % for optimized structures, indicating a strong magneto-optical response. This level of response represents a significant advancement compared to the values typically observed in nanostructured arrays that do not incorporate a GT cavity. For example, studies exploring the interaction between plasmonic chiral oligomers (chiral Au structures with an MgF_2_ film) and Au/Co multilayers reported magneto-optical Δ*T* (differential transmittance) values around −0.3 %–0.6 % [22]. Similarly, systems based on 2D composite trimer nanoantennas, which were foundational in demonstrating dynamic real-time modulation of chiroptical response, reported a field-induced absorbance modulation (Δ*A*) of −0.3 % [8]. Furthermore, important work focused on the methodological challenge of isolating pure MCD signals from substrate-induced artefacts (using a 50 nm Co film on polymer tape) successfully identified an intrinsic MCD (Δ*T*) of 1.2 % [45].

## 4 Modulators based on magneto-optical metasurfaces

**Figure 11:**
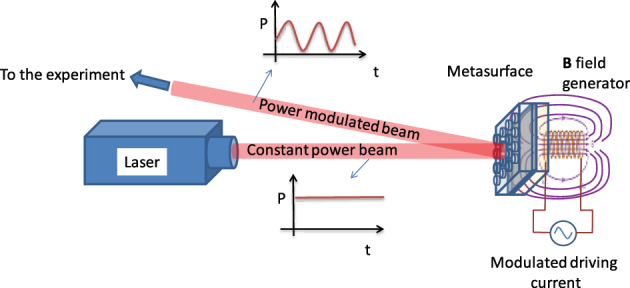
Metasurface-based intensity modulation setup. Schematic of the proposed integration of the GT metasurface as an intensity modulator.

To explore the potential application of magnetic tunability in the proposed systems, we focus our attention on disk-shaped meta-atom metasurfaces. The metasurfaces with *P* = 0.280 µm and *P* = 0.500 µm exhibit significant response to an external magnetic field for different underlying reasons. In the case of the *P* = 0.280 μm GT metasurface, the enhanced magnetic response arises from a combination of factors: the high density of ferromagnetic meta-atoms, which facilitates stronger magneto-optical interactions, and the presence of hybridization processes, as shown in Figures 13S and 24S of the Supporting Information. Conversely, in the case of the GT metasurface with *P* = 0.500 μm, the magneto-optical properties mainly arise from the couplings between LSPR, SLR, and GT cavity modes. The density of ferromagnetic meta-atoms contributes to a lesser extent in this case compared to the *P* = 0.280 µm metasurface. We propose this system as the basis for an amplitude modulator under the influence of an external magnetic field, with potential integration with a laser source as shown in Figure 11.

The metasurface, placed within the optical path, modulates the intensity of reflected light based on the applied external magnetic field, with distinct responses for RCP and LCP light. It must be noticed that the response for LCP and RCP light can be exchanged by inverting the direction of the magnetic field with respect to the metasurface. Therefore, full amplitude modulation can be achieved on a single impinging polarization handedness by inverting the sign of the magnetic field. This configuration enables tunable reflectance and is designed for potential integration into laser systems, offering precise control over light intensity and polarization.

For this study, we selected a wavelength in the near-infrared region, specifically 0.900 µm. To achieve the maximum difference in reflectance between RCP and LCP light while maintaining an acceptable average reflectance, we carefully refined and optimized again the structural parameters to have maximum magneto-optical effect at a wavelength of 0.900 µm (see also Supplementary Information). The starting design parameters selected for refinement were the most promising metasurfaces among the ones described in the previous sections, characterized by *P* = 0.280 μm and *P* = 0.500 μm.

The optimized parameters for the metasurface with *P* = 0.280 µm yielded a magnetic field induced reflectance contrast of Δ*R*/*R* = 23.0 % between RCP and LCP light as shown in Table 1.

**Table 1:** Structural and reflectance parameters (S.P. and R.P.).

S.P.	Value	R.P.	Value
*P*	0.280 μm	$R_{\text{RCP}}\left(\lambda \right)$	24.2 %
*t*_*d*_	0.510 μm	$R_{\text{LCP}}\left(\lambda \right)$	30.5 %
*t*_*m*_	0.012 μm	Δ*R*	6.3 %
*R*	0.120 μm	Δ*R*/*R* %	23.0 %

For the metasurface with an initial periodicity of 0.500 µm, we obtained the refined structural parameters and the optical behavior reported in Table 2.

**Table 2:** Structural and reflectance parameters (S.P. and R.P.).

S.P.	Value	R.P.	Value
*P*	0.450 μm	$R_{\text{RCP}}\left(\lambda \right)$	18.4 %
*t*_*d*_	0.480 μm	$R_{\text{LCP}}\left(\lambda \right)$	23.6 %
*t*_*m*_	0.020 μm	Δ*R*	5.2 %
*R*	0.140 μm	Δ*R*/*R* %	24.8 %

The optimized parameters for the metasurface with initial *P* = 0.500 µm, listed in Table 2, yielded a magnetic field induced reflectance contrast of Δ*R*/*R* % = 24.8 % between RCP and LCP light.

In a practical device, the known tendency of Co to oxidize should be avoided. For this purpose, we simulated the addition of a SiO_2_ protective layer (25 nm) to the optimized structure from Table 2 (*P* = 0.450 μm, *t*_*d*_ = 0.480 μm, *t*_*m*_ = 0.020 μm, *R* = 0.140 μm). This passivated, robust structure achieves a maximum absolute reflectance percentage difference (Δ*R*) of 5.6 %, which is even slightly larger than Δ*R* = 5.2 % calculated for the identical structure without the protective layer. This demonstrates that the necessary protection for practical device implementation is fully compatible with the enhanced magneto-optical effects achieved in our design.

These results make these metasurfaces suitable for practical applications such as a high-speed external amplitude modulator for a laser source as schematically shown in Figure 11.

## 5 Conclusions

In this work, we propose a metasurface able to achieve magnetic field-dependent optical response. We demonstrate how the interplay between magnetoplasmonic modes of ferromagnetic nanostructures, GT cavity modes, and surface lattice resonance modes arising from the nanodisks periodic arrangement can provide a distinct advantage in achieving tunable effects, even with relatively low external magnetic fields, compared to the cavity without meta-atoms and unstructured systems. We highlight the significant role of meta-atom shape and periodicity in controlling the magneto-optical response of the systems. We demonstrate that the use of nanostructured systems, such as metasurfaces, offers enhanced opportunities for tunability by allowing precise manipulation of structural factors in the design. Specifically, we introduce a GT metasurface design that operates as an intensity modulator, with potential applications for integration into laser sources. This system exhibits distinct responses to RCP and LCP light under the influence of an external magnetic field, achieving a maximum reflectance contrast of 24.8 % between the two polarization states. It must be noticed that previous experiments of MOKE effect on nanostructured arrays of ferromagnetic meta-atoms usually show Kerr rotation angles for linear polarization of the order of 10–20 mrad [43], [46], [47], which would roughly correspond to reflectivity contrast for LCP/RCP polarization of ∼1–2 %. This confirms that the use of the GT resonant cavity enables a significant enhancement of MOKE effects with respect to ordered arrays alone.

Looking forward, we aim to further explore emerging trends in materials for magneto-optics and magnetoplasmonics [16], [48], [49], [50] such as the use of conductive oxide nanostructures [51], [52] as potential building blocks for metasurfaces design. Such structures typically exhibit much sharper LSPR resonances, which are expected to result in larger effects when coupled to Fabry–Pérot cavity or surface lattice modes.

We believe this study can trigger significant future advancements of magnetoplasmonic modulation in areas such as imaging, sensing, and communications. In particular, compact designs facilitated by structured GT cavities allow for miniaturized and scalable devices in integrated photonics and sensing, such as on-chip optical isolators, wearable magnetic field sensors, and lab-on-a-chip systems. Metamaterial advancements enable the control via magnetic field of structured surfaces, driving innovations in active and adaptive optics for applications like beam steering, light shaping, and programmable photonic devices.

## Supplementary Material

Supplementary Material Details
