# Supplementary material for: Synergistic enhancement of magneto-optical response in cobalt-based metasurfaces via plasmonic, lattice, and cavity modes
Source: Nanophotonics. 2025 Dec 9;14(27):5511–25. doi: 10.1515/nanoph-2025-0495 (PMC12717927; doi:10.1515/nanoph-2025-0495)
Supplement: Supplementary file 1 — Supplementary Material Details [file j_nanoph-2025-0495_suppl_001.docx]

**Supporting Information**

**Synergistic Enhancement of Magneto-Optical Response in Cobalt Based Metasurfaces via Plasmonic, Lattice, and Cavity Modes.**

Alberto Santonocito^1,2^, Alessio Gabbani^2,3^, Barbara Patrizi^1^, Guido Toci^1, *^ and Francesco Pineider^2, *^

*^1^*Istituto Nazionale di Ottica (INO), Consiglio Nazionale delle Ricerche (CNR), Via Madonna del Piano 10, 50019, Sesto Fiorentino (FI), Italy

*^2^*Dipartimento di Chimica e Chimica Industriale, Università di Pisa, Via Moruzzi 13, 56124, Pisa, (Italy)

*^3^*Dipartimento di Chimica “Ugo Schiff”, Università di Firenze, Via della Lastruccia 13, 50019, Sesto Fiorentino (FI), Italy

E-mail:

[francesco.pineider@unipi.it](mailto:francesco.pineider@unipi.it)

guido.toci@ino.cnr.it

**a. Definitions and conventions for dielectric permittivity**

To determine the dielectric permittivity of Co and Al, their respective refractive indices are utilized in the following relation:

$\varepsilon_{r}=n^{2}-k^{2}$ and $\varepsilon_{im}=2nk$ (1)

then, $\varepsilon$ is obtained by using the complex equation:

$\varepsilon=\varepsilon_{r}-i\varepsilon_{im}$ (2)

In the context of Equations (1) and (2), $n$ and $k$ represent the real part and the imaginary part (extinction coefficient) of the complex refractive index, while $\varepsilon_{r}$ and $\varepsilon_{im}$ are the real (dispersive) and imaginary (absorptive) parts of the dielectric function $\varepsilon$, respectively.

b. Polar magneto-optical Kerr Effect (p-MOKE)

The following equations summarize the p-MOKE:

$\varphi_{K}= -\left( \frac{\varphi_{RCP} - \varphi_{LCP}}{2} \right)$ (3)

$e_{K} =\frac{(r_{RCP} - r_{LCP})}{\left( r_{RCP} + r_{LCP} \right)}$ (4)

where $\varphi_{K}$ is the phase variation between RCP and LCP lights, $e_{K}$ is the ellipticity and $r_{RCP}$ and $r_{LCP}$ are the Fresnel coefficients.

The Fresnel coefficients (complex reflectance amplitudes) for RCP and LCP light are given by:

$r_{RCP} =\frac{(n_{RCP} - 1)}{\left( n_{RCP} + 1 \right)}$ (5)

$r_{LCP} =\frac{{(n}_{LCP} - 1)}{\left( n_{LCP} + 1 \right)}$ (6)

The reflectance for RCP and LCP light is related to the Fresnel coefficients through the equations:

$R_{RCP}= {|r_{RCP}|}^{2}$ (7)

and

$R_{LCP}= {|r_{LCP}|}^{2}$ (8)

Equations (9) and (10) describe the effect of an external magnetic field on the squared refractive index of a magneto-optical material when interacting with RCP and LCP light.

${n_{RCP}^{2} =\varepsilon}_{xx}+{i\varepsilon}_{xy}$ (9)

${n_{LCP}^{2} =\varepsilon}_{xx}-{i\varepsilon}_{xy}$ (10)

where $n_{RCP}^{2}$ and $n_{LCP}^{2}$ are the square of the refractive index of a magneto-optical material and $\varepsilon_{xy}$ is the off diagonal term of the dielectric permittivity tensor. These effects can be understood by decomposing the incident LP light into right-circularly polarized (RCP) and left-circularly polarized (LCP) components (see Figure 1S).

**Figure 1S**: (a) LP light represented as the superposition of RCP and LCP components with equal amplitude and same modulus of phase velocity, but opposite direction of rotation. (b) Rotation of LP light due to a difference in phase velocities modulus between RCP and LCP. (c) General case showing elliptical polarization arising from both differential absorption and phase velocity modulus between RCP and LCP components.

**c. Isolated Co disk**

When considering an isolated ferromagnetic meta-atom, it is already reported that the external magnetic field can modulate the Localized Surface Plasmon Resonances (LSPR)[1], [2], [3], [4], [5], thus giving rise to a relatively large magneto-optical response. The Lorentz force generated by the magnetic field interacts with the oscillating charge density, resulting in a shift of these resonances towards either longer or shorter wavelengths (Figure 2S (**a**)-(**b**)). To further illustrate the polarization-dependent optical response of a ferromagnetic meta-atom, we examine the effects of the external magnetic field on an isolated Co disk with a radius *R* of 120 nm and a height *h* of 15 nm. Specifically, we investigate the distribution of the electric field norm (Figure 2S (**c**)-(**d**)), and the extinction cross section in the presence of an external magnetic field and RCP or LCP incoming light (Figure 3S).


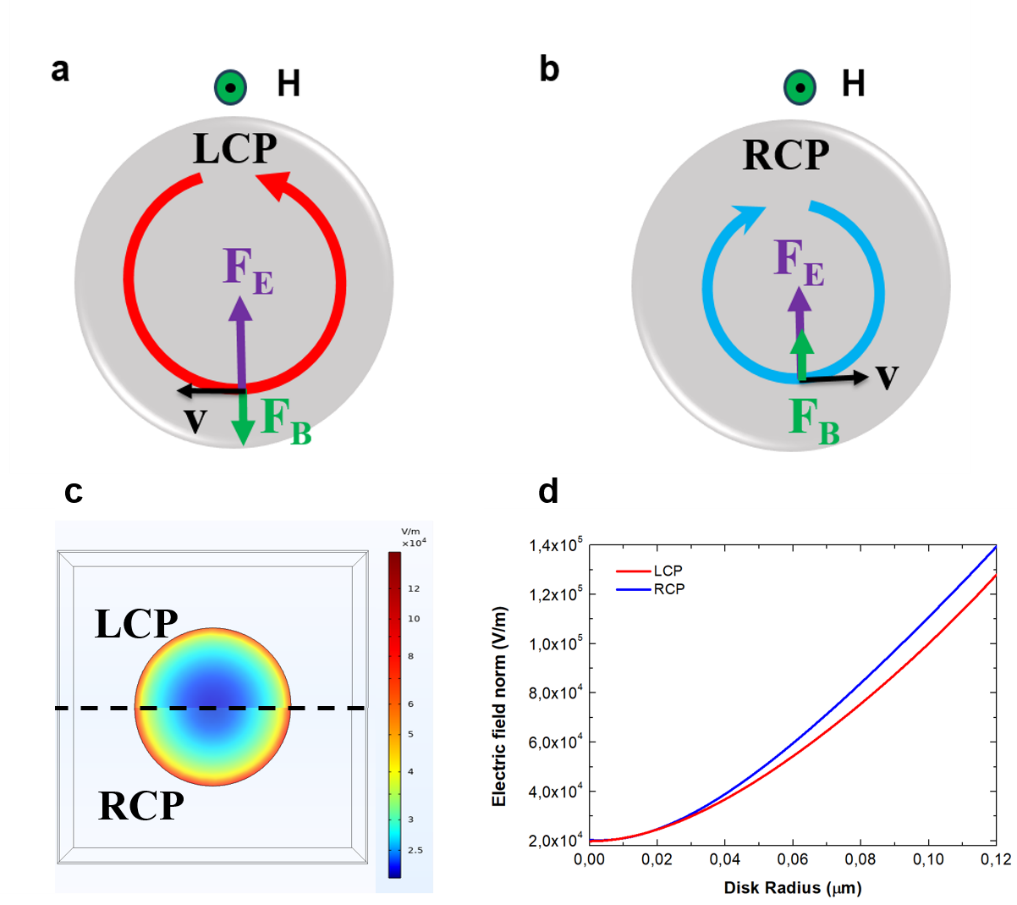


**Figure 2S: (a)** Illustration of the effects of the magnetic force **F_B_** and the electric force **F_E_** (together constituting the Lorentz force) on an electron with velocity **v** associated with the oscillating electron density on the meta-atom surface in the presence of LCP. The convention of arrows is employed, with the green dot indicating a magnetic field exiting the plane of the page. **(b)** Similar illustration for an electron with velocity **v** on the meta-atom surface under RCP. (**c**) False color graph depicting the electric field norm on the disk surface, illustrating the spatial variation in electric field intensity, and (**d**) a plot of the electric field norm along the disk radius, showing the effect of the disk radius.


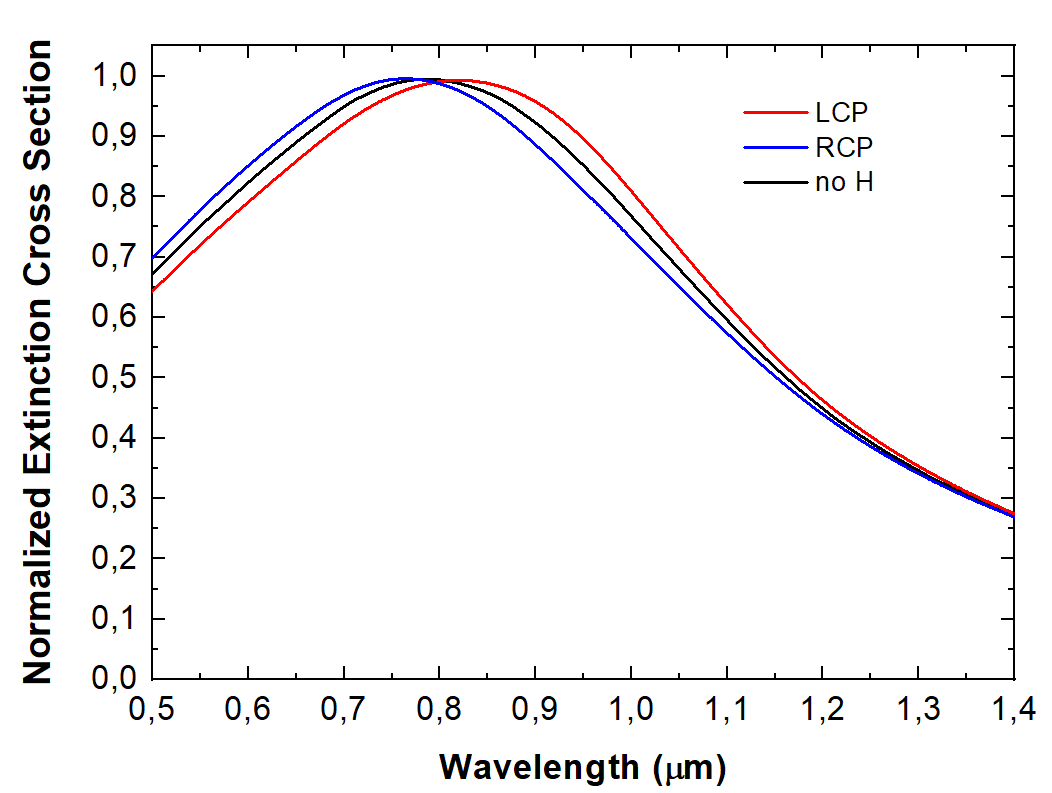


**Figure 3S**: Extinction Cross section of an isolated Co disk (*R* = 120 nm and *h* = 15 nm) without an external magnetic field (black line), and in the presence of an external magnetic field with RCP interacting light (blue line) or LCP interacting light (red line).

Figure 3S displays the extinction cross sections of the Co disk in the LSPR range under three distinct conditions: without an external magnetic field (black line), and in presence of an external magnetic field with RCP light (blue line) and LCP light (red line).

In the absence of external magnetic field, the responses for LCP and RCP coincide. On the other hand, the LSPR shifts under an external magnetic field (here set at a value of 0.67 T, corresponding to the experimental data reported in reference [6]). The field-induced shift is due to the Lorentz force acting on charge carriers (Figure 2S). The spectral characteristics are strongly influenced by LSPR. Upon interaction with a plasmonic nanoparticle, incident electromagnetic radiation can undergo absorption and scattering. Absorption involves the conversion of photons into heat within the nanoparticle, whereas scattering redirects photons in different directions. The efficiencies of these processes can be quantified by considering the absorption and scattering cross sections. At the resonance peak, the absorption and scattering cross sections of a plasmonic nanoparticle can significantly exceed its physical size, allowing it to interact with light from an effective larger area.

**d. Scattering albedo**

In the context of discrete particles interacting with incident light, we can express the total intensity of the incident light ($I_{in}$*)* as the sum of the absorbed intensity ($I_{abs}$), the scattered intensity ($I_{sc}$), and the transmitted intensity ($I_{t}$). This relationship is formalized in the following equation:

$I_{in}=I_{abs}+I_{sc}+I_{t}$ (11)

In this equation, $I_{t}$ is considered to be the non-interacting portion of the intensity, while the interacting fraction comprises the absorbed ($I_{abs}$) and scattered intensities ($I_{sc}$). Notably, $I_{sc}$ accounts for both forward-scattering and back-scattering contributions. These concepts are represented in Figure 4S.


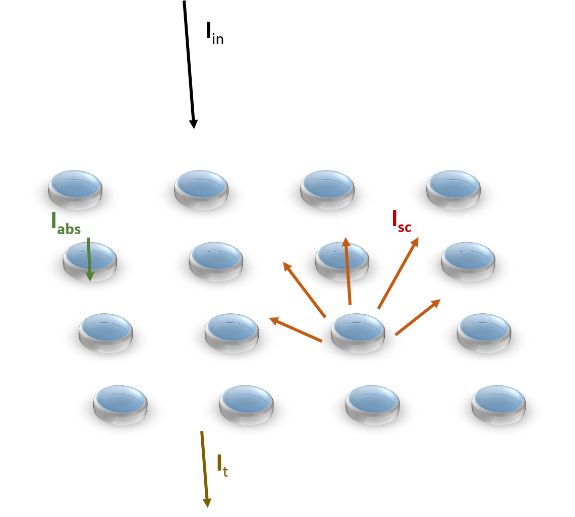


**Figure 4S**: Physical phenomena implicated when an incident electromagnetic field interacts with an array of discrete particles.

The scattering albedo ($\sigma_{\mathrm{alb}}$) is the ratio between $I_{\mathrm{sc}}$ and $I_{\mathrm{abs}}+I_{\mathrm{sc}}$ (i.e. the total interacting intensity).

$\sigma_{\mathrm{alb}}=\frac{I_{\mathrm{sc}}}{I_{\mathrm{sc}}+I_{\mathrm{abs}}}$ (12)

we do not consider I_t_ because is the non-interacting fraction of the intensity.

By substituting $I_{\mathrm{sc}}=I_{\mathrm{in}}\sigma_{\mathrm{sc}}$ and $I_{\mathrm{abs}}=I_{\mathrm{in}}\sigma_{\mathrm{abs}}$ into the albedo expression, we obtain:

$\sigma_{\mathrm{alb}}=\frac{\sigma_{\mathrm{sc}}}{\sigma_{\mathrm{sc}}+\sigma_{\mathrm{abs}}}$ (13)

For a continuous plane we have that $I_{\mathrm{in}}$ must interact, thus, in this case the scattering cross section is equal to:

$\sigma_{\mathrm{sc}}=\frac{I_{t}+I_{sc}}{I_{\mathrm{in}}}$ (14)

Figure 10S graphically illustrates these case.


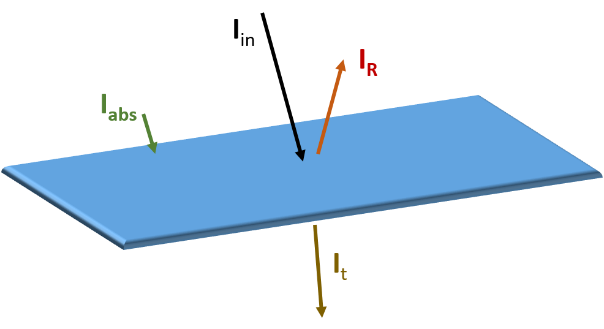


**Figure 5S:** continuous plane interacting with an incident electromagnetic field.

In this framework, the scattering albedo could be expressed as:

$\sigma_{\mathrm{alb}}=\frac{I_{R}+I_{t}}{I_{R}+I_{t}+I_{\mathrm{abs}}}=\frac{I_{\mathrm{in}}(R+T)}{I_{\mathrm{in}}(R+A+T)}$ (15)

where R is the reflectance, A is the absorbance, and T is the transmittance.

If the surface is sufficiently opaque such that $I_{t}=0$, the scattering albedo become:

$\sigma_{\mathrm{alb}}=R$ (16)

This simplification matches with the fundamental principle that the sum of reflectance (R), absorbance (A), and transmittance (T) must equal one ($R+A+T=1$).

**e. Co disk on a SiO_2_ layer**

We also performed simulations of the extinction cross section for the same Co disk (*R* = 120 nm and *h* = 15 nm) positioned on a bulk layer of SiO_2_. This analysis allows us to estimate the shift in the position of the Co LSPR due to the influence of the underlying substrate.

Figure 6S reports the normalized scattering (σ_sc_), absorption (σ_abs_) and extinction (σ_ext_) cross sections for this system in comparison with the isolated disk.

~~
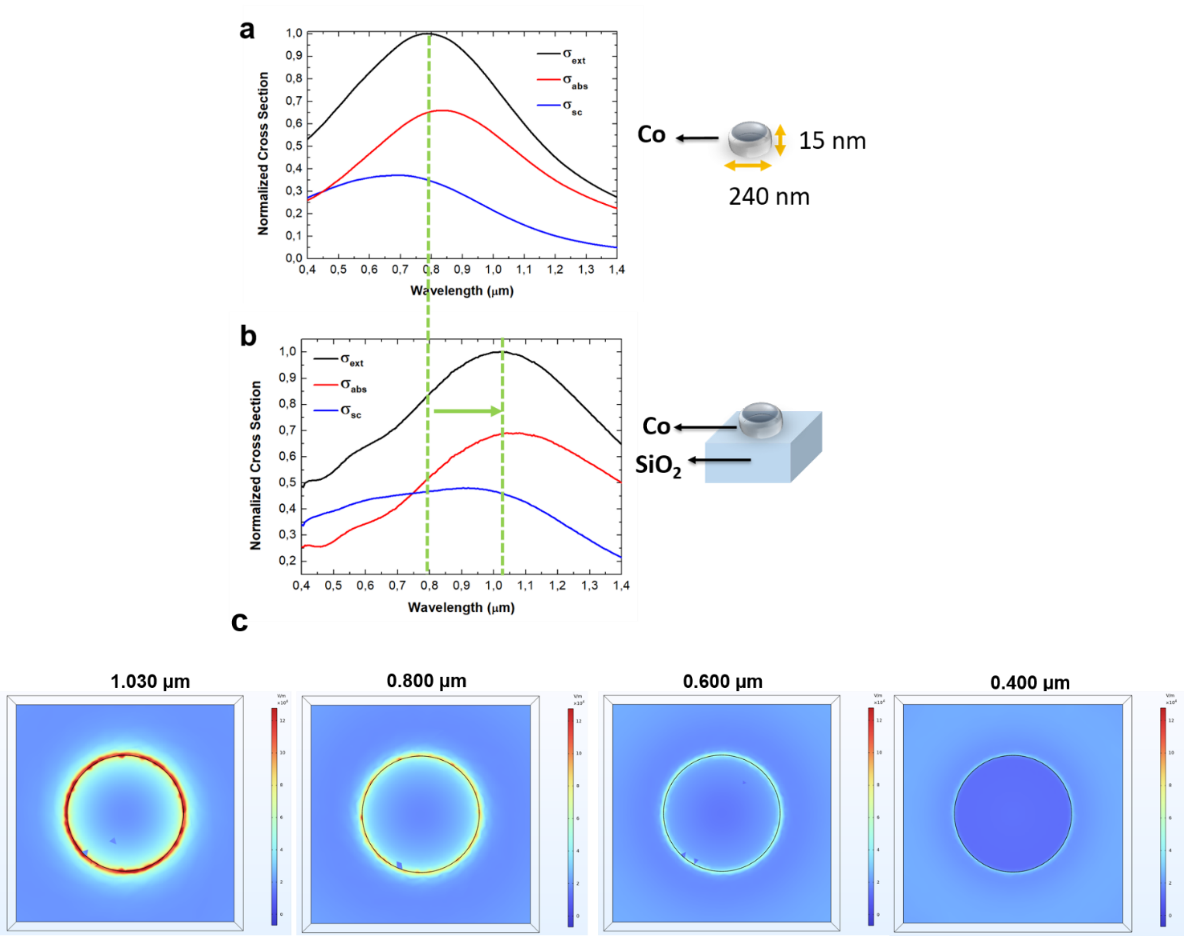
~~

**Figure 6S**: σ_sc_, σ_abs_ and σ_ext_ of the isolate Co disk (*R* = 120 nm and *h* = 15 nm) (**a**) and σ_sc_, σ_abs_ and σ_ext_ of the isolate Co disk (*R* = 120 nm and *h* = 15 nm) atop a SiO_2_ substrate (**b**). The green dashed lines highlight the shift due to the presence of SiO_2_ substrate. (**c**) Electric field distribution at different wavelengths, illustrating the LSPR mode and its gradual intensity decrease toward shorter wavelengths.

Figure 6S shows a redshift of the extinction peak occurs when the Co disk is placed on a SiO_2_ bulk layer (Figure 6S (**b**)) with respect to when it is surrounded by air (Figure 6S (**a**)). This redshift can be attributed to the changes in the local refractive index around the Co disk due to the presence of the SiO_2_ substrate.

Figure 7S presents the spectra of the scattering albedo (that, for isolated particles, corresponds to diffuse reflectance for continuous surfaces), for both RCP and LCP lights, along with their differential spectrum. The scattering albedo ($\sigma_{alb}$) was calculated by the ratio of $\sigma_{sc}$ and $\sigma_{ext}$ as reported in the following relation:$\sigma_{alb} =\frac{\sigma_{sc}}{\sigma_{ext}}$ (see Section d of Supporting Information for further details on $\sigma_{alb}$ and its comparability to the reflectance of continuous systems).

It can be observed that the disparities between scattering albedo values decrease as the wavelength increases, with the maximum difference of 0.036 at 0.450 μm.


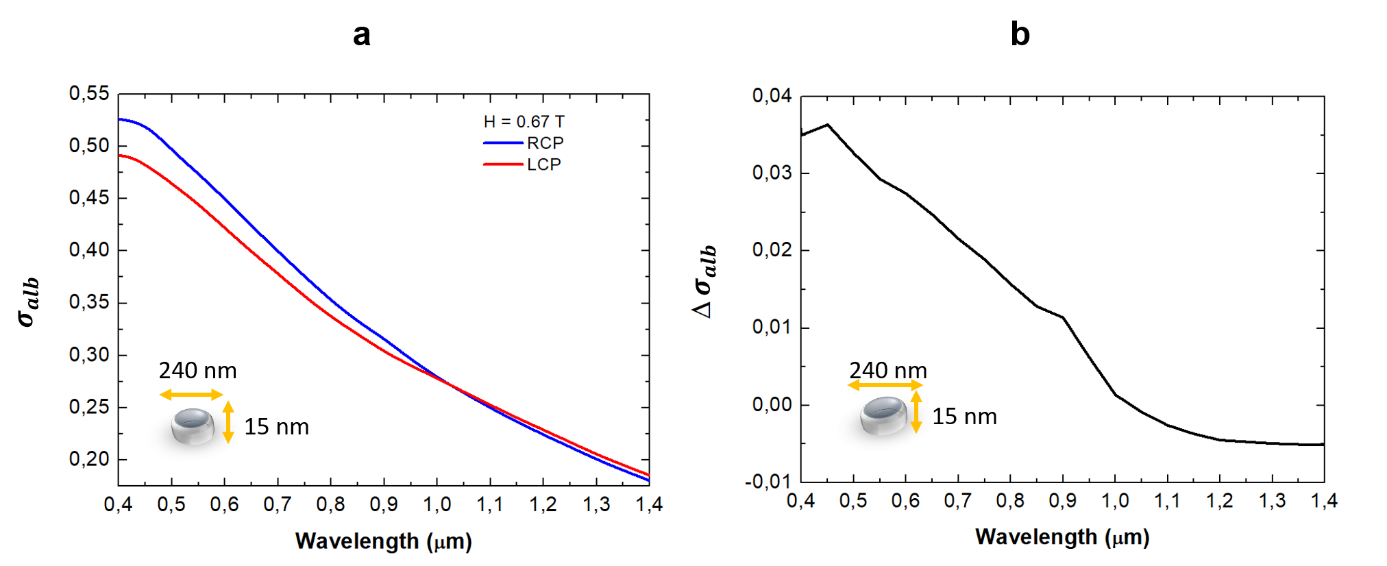


**Figure 7S:** (**a**) Comparison of the scattering albedo spectra for the isolated disk in air interacting with RCP (blue line) and LCP (red line) lights. (**b**) Differential spectrum illustrating the reflectance differences between RCP and LCP polarizations.

**f. Infinitely extended Co thin layer**

The second case examined is presented in Figure 8S, which illustrates the reflectance spectra for RCP and LCP light from an infinitely extended cobalt layer. The figure also includes the differential spectrum, highlighting the differences between the reflectance spectra of the two polarization states. Again, a decreasing trend in the difference between reflectance spectra is observed as wavelength increases, with a maximum difference of about 0.058 at 0.400 μm.


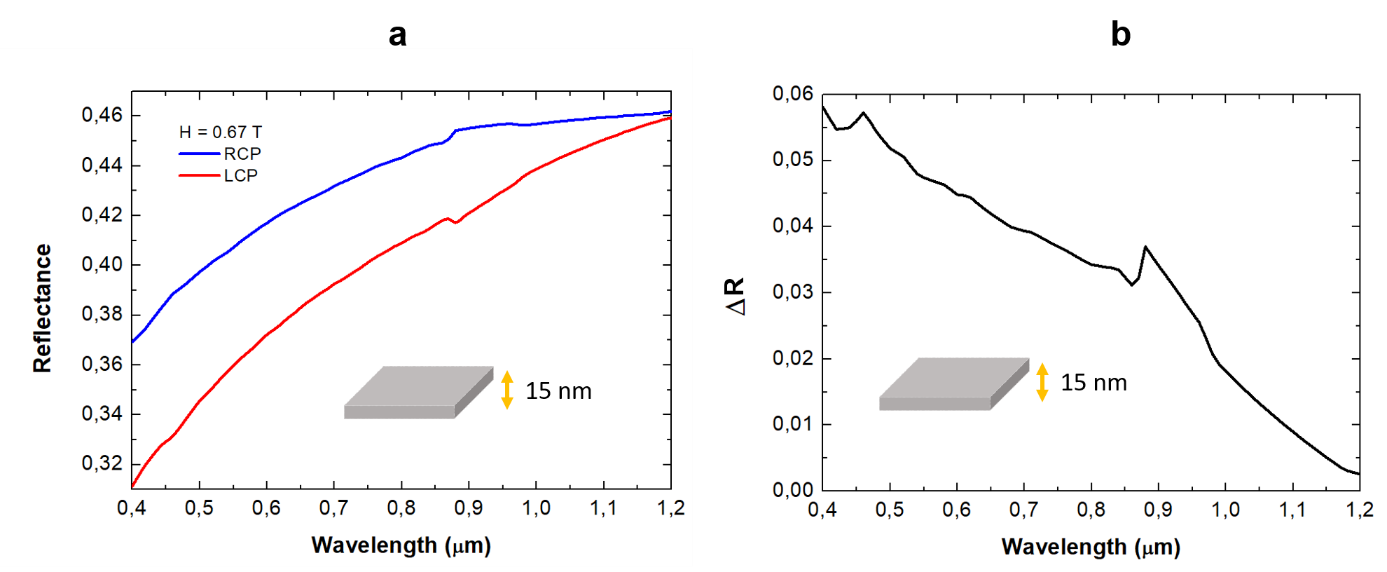


**Figure 8S:** (**a**) Comparison of the reflectance spectra for the Co infinitely extended layer interacting with RCP (blue line) and LCP (red line) lights. (**b**) Differential spectrum illustrating the reflectance disparity between RCP and LCP polarizations.

We also observed small phase variations for both isolated systems. In Figure 9S we report, as an example, the phase delay for RCP and LCP lights for an infinitely extended Co thin layer.


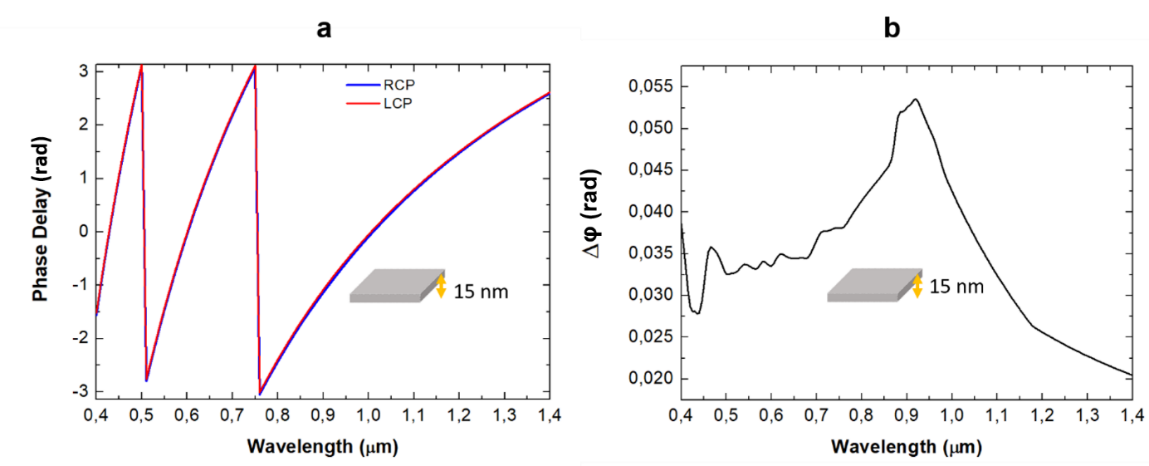


**Figure 9S:** Phase delay analysis for RCP (blue line) and LCP (red line) incident lights on a continuous Co layer.

Figure 9S indicate that the amplitude and phase variations induced by the p-MOKE are not particularly remarkable in these isolated ferromagnetic systems.

**g. Infinitely extended GT resonator**

To further investigate the ferromagnetic response of the infinitely extended Co layer in more complex structures, we incorporated it into a multilayered system made of a SiO_2_ layer, a Co layer, and an Al substrate, effectively forming a high-loss GT cavity bounded by the dielectric-metal interface and by the air-dielectric interface. The SiO_2_ thickness was set to 0.5 µm.

Simulation of the reflectance spectrum for this structure reveal pronounced dips at wavelengths of 0.44 µm, 0.60 µm, and 0.98 µm (corresponding to GT cavity modes), as shown in Figure 10S(**a**). Additionally, Figure 10S (**b**-**c**) displays reflectance and phase variations for the same multilayered system.


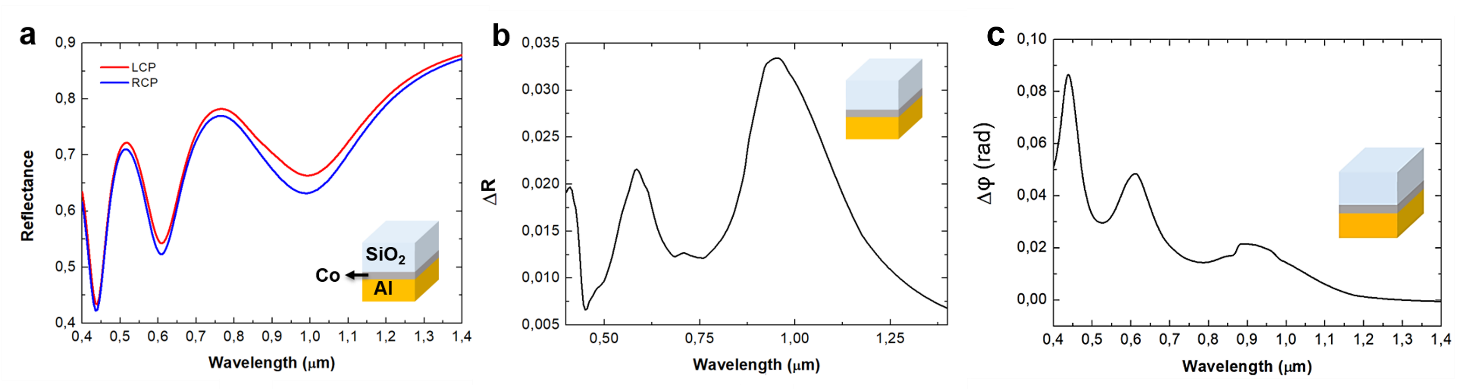


**Figure 10S**: (**a**) Reflectance spectra for a multilayer system (SiO_2_/Co/Al) interacting with LCP (red line) and RCP (blue line) lights. (**b**) and their difference. (**c**) Differential phase delay spectra highlighting the variations in phase delay between LCP (red line) and RCP (blue line) lights.

**h. MIM magneto-optical metasurfaces**

The simulated system comprises a 0.015 μm thick Co disk with a diameter of 0.24 μm, placed on top of a 0.5 μm thick silica layer. Beneath the silica layer is another 0.015 μm thick Co layer, situated above a reflective bulk Al substrate.


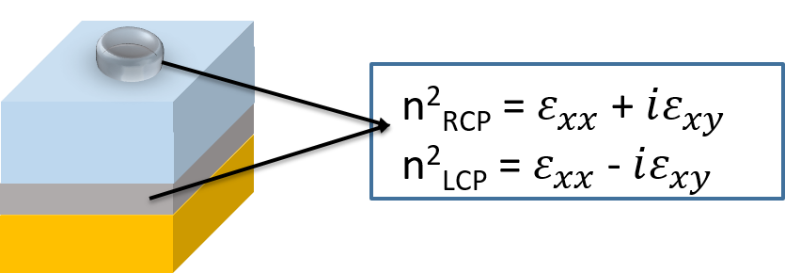


**Figure 11S**: Modification of the cavity modes boundaries with the application of an external magnetic field (H).

Figure 12S (**a**-b) illustrates the reflectance spectra for RCP and LCP light resulting from a metasurface with a spacing between the meta-atoms *P* = 1µm. The differential spectrum between RCP and LCP reflectances is also shown. Additionally, Figure 12S (**c**) displays the differential phase delay between them.


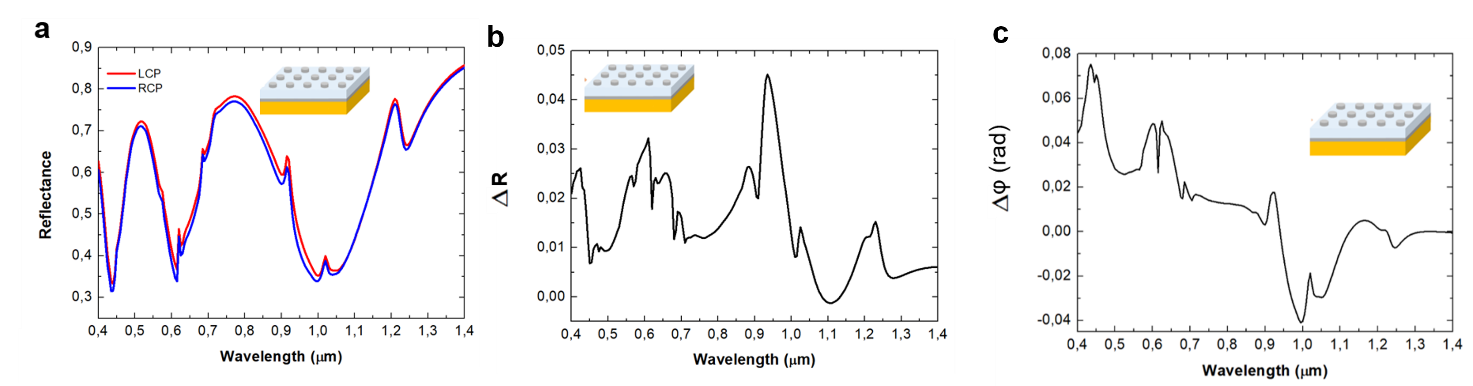


**Figure 12S**: Metasurface with meta-atom spacing *P* = 1 µm. (**a**) Comparison of the reflectance spectra for the simulated MIM GT metasurface with RCP (blue line) and LCP (red line) lights. (**b**) Differential reflectance spectrum illustrating the reflectance differences between RCP and LCP polarizations. (**c**) Differential phase delay spectra highlighting the variations in phase between RCP and LCP lights.

The metasurface in Figure 12S presents a more complex pattern due to multiple couplings between cavity modes and other phenomena such as LSPRs and grating diffraction.

Concerning the phase delay for RCP and LCP lights, as well as the differential phase delay between them, our analysis indicates that the differences are more pronounced at a spacing *P=* 1 μm compared to the (SiO_2_/Co/Al) GT resonator, as shown in Figure 10S.

In Figures 13S and 14S, we present the reflectance spectra for metasurfaces featuring different meta-atom geometries. In Figure 13S, the metasurface consists of disk-shaped meta-atoms with a period (P) of 0.280 μm, while in Figure 14S, the metasurface is composed of square-shaped meta-atoms, with the same period.


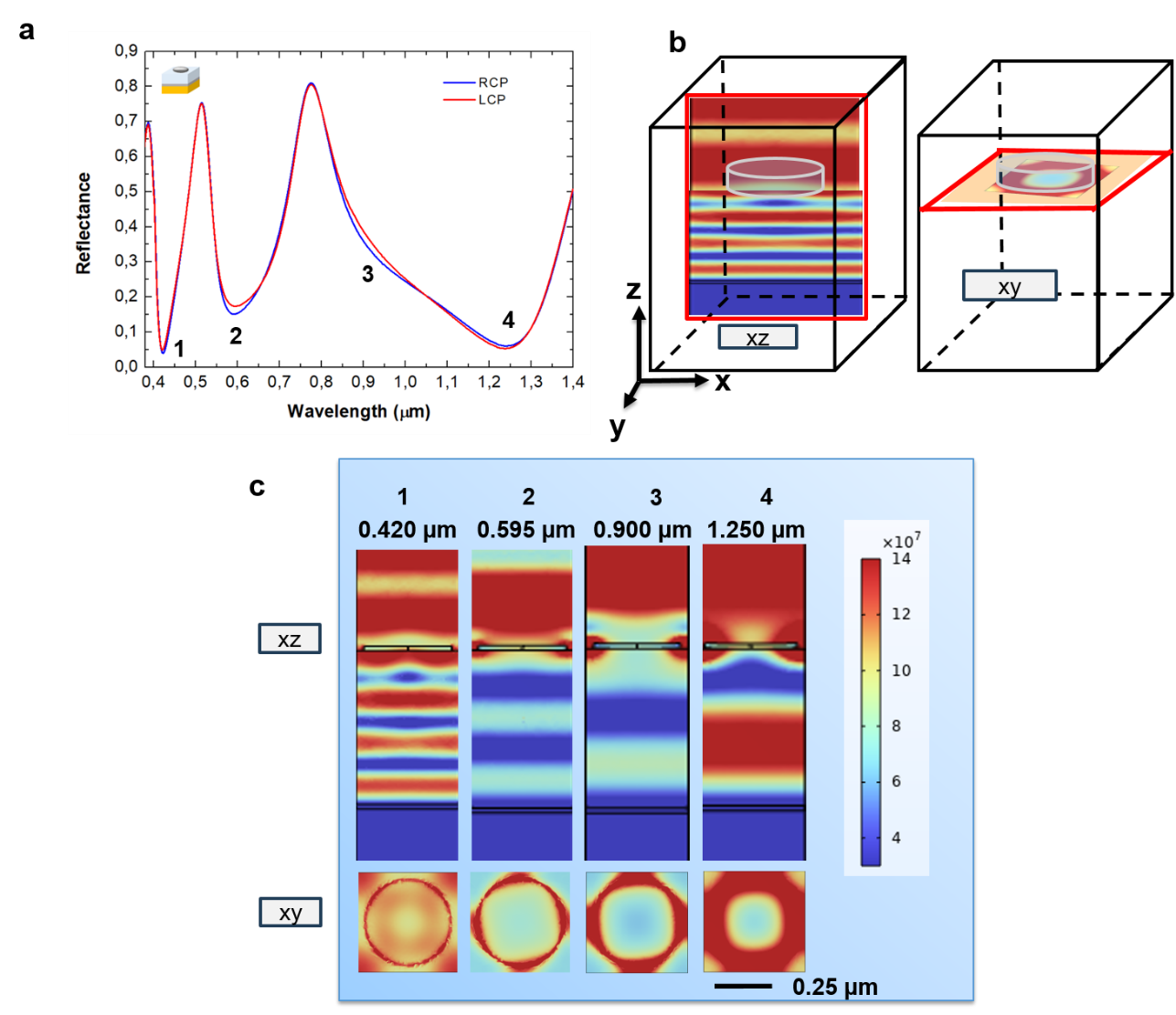


**Figure 13S**: Metasurface with disk-shaped meta-atoms and *P* = 0.280 µm. Comparison of the reflectance spectra for the simulated MIM GT meatsurface with RCP (blue line) and LCP (red line) lights.


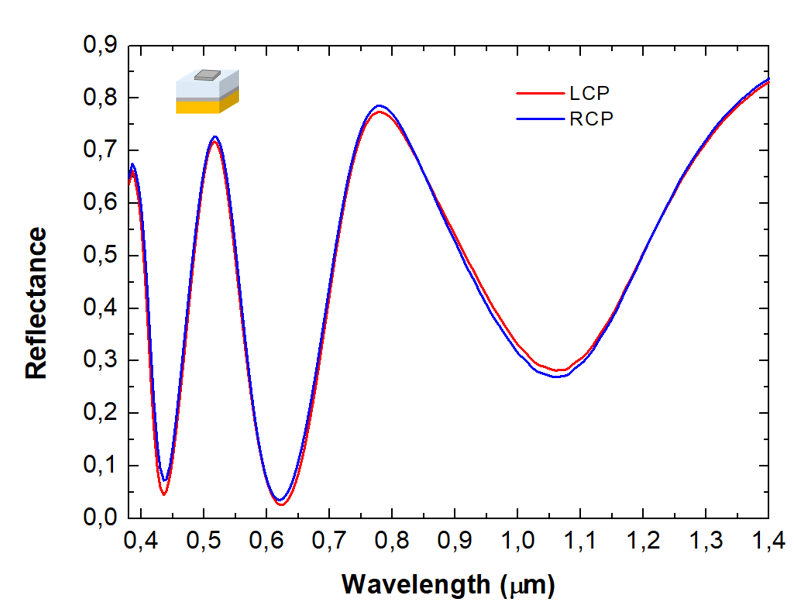


**Figure 14S**: Metasurface with square meta-atoms and *P* = 0.28 µm. Comparison of the reflectance spectra for the simulated MIM GT meatsurface with RCP (blue line) and LCP (red line) lights.

In Figure 13S (**c**) we observe that a surface wave anomaly (Wood anomaly) occurs at 0.420 µm. This anomaly is predicted by Equation (4) in the paper (or Equation (19) here, in Supporting Information) when using the effective refractive index $n= \left( \frac{\varepsilon_{Co}\varepsilon_{{SiO}_{2}}}{\varepsilon_{Co}+\varepsilon_{{SiO}_{2}}} \right)^{\frac{1}{2}}$ and $P= \sqrt{2}\cdot0.280 \mu m$.

**i. Trend of the magneto-optical response with structural parameters**

We investigate the magneto-optical response, specifically the reflectance and phase variation for RCP and LCP light, under the influence of an external magnetic field. This analysis is conducted by varying the structural parameters of the Gires-Tournois (GT) metasurface composed of disk-shaped meta-atoms. In Figure 15S and 16S we report the differential spectra of reflectance and phase for RCP and LCP light varying only the thickness of the Co disks and Co coating layer, while keeping the following parameters fixed: 𝑡_d_ = 0.5 𝜇𝑚, 𝑅 = 0.12 𝜇𝑚, 𝑃 = 0.28 𝜇𝑚, and 𝜆 = 0.9𝜇𝑚.


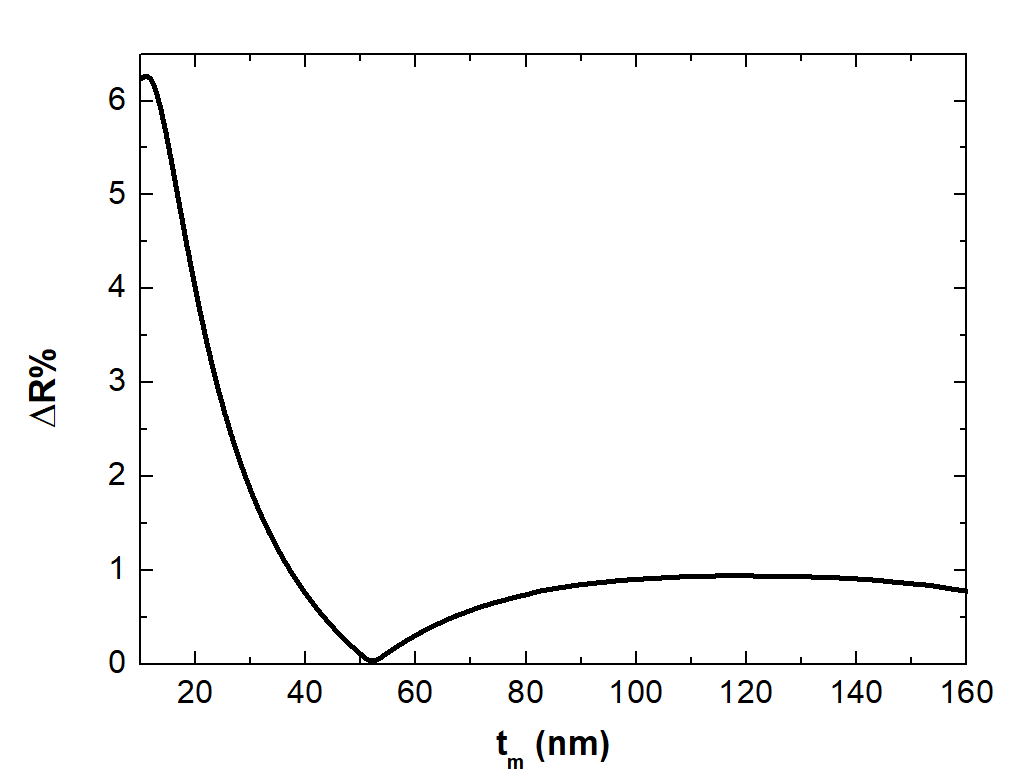


**Figure 15S**: Differential reflection between RCP and LCP light as a function of the thickness (t_m_) of the disk-shaped meta-atoms and the cobalt (Co) layer.


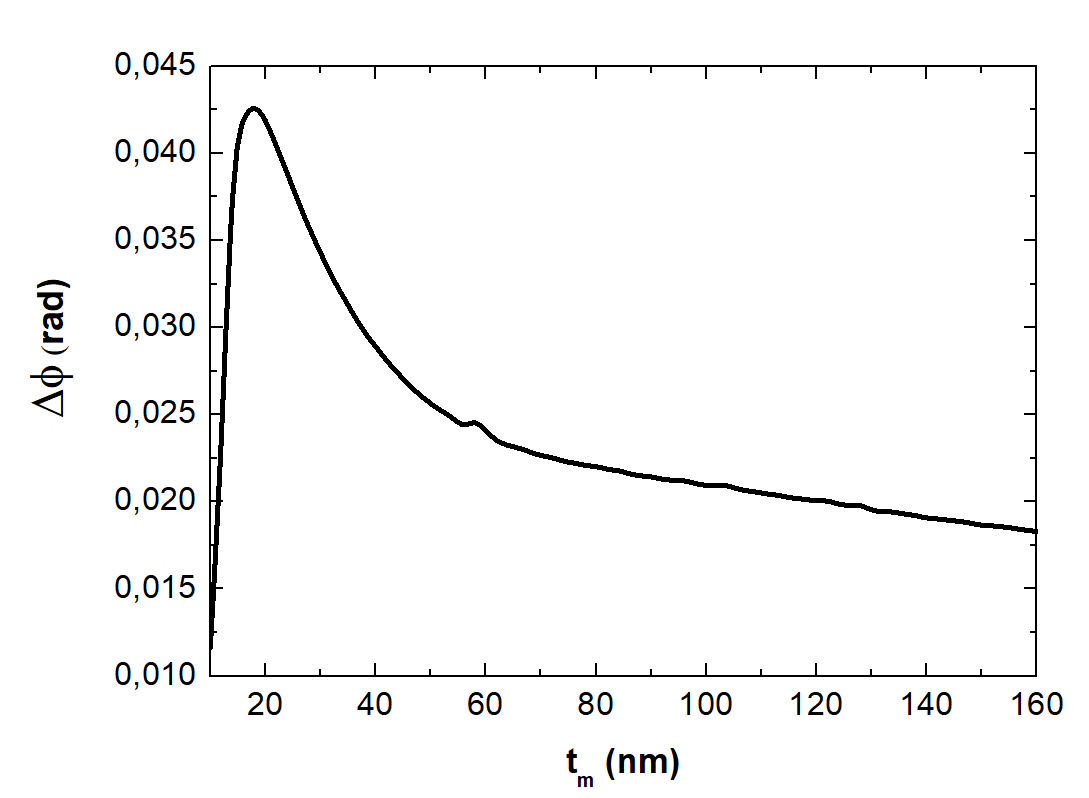


**Figure 16S**: Differential phase between RCP and LCP light as a function of the thickness (t_m_) of the disk-shaped meta-atoms and the cobalt (Co) layer.

In Figures 17S and 18S, we report the differential spectra of reflectance and phase for LCP and RCP light, varying the radius while keeping the following parameters fixed: 𝑡_d_ = 0.5 𝜇𝑚, 𝑡_m_ = 0.012 𝜇𝑚, 𝑃 = 0.28 𝜇𝑚, and 𝜆 = 0.9𝜇𝑚.


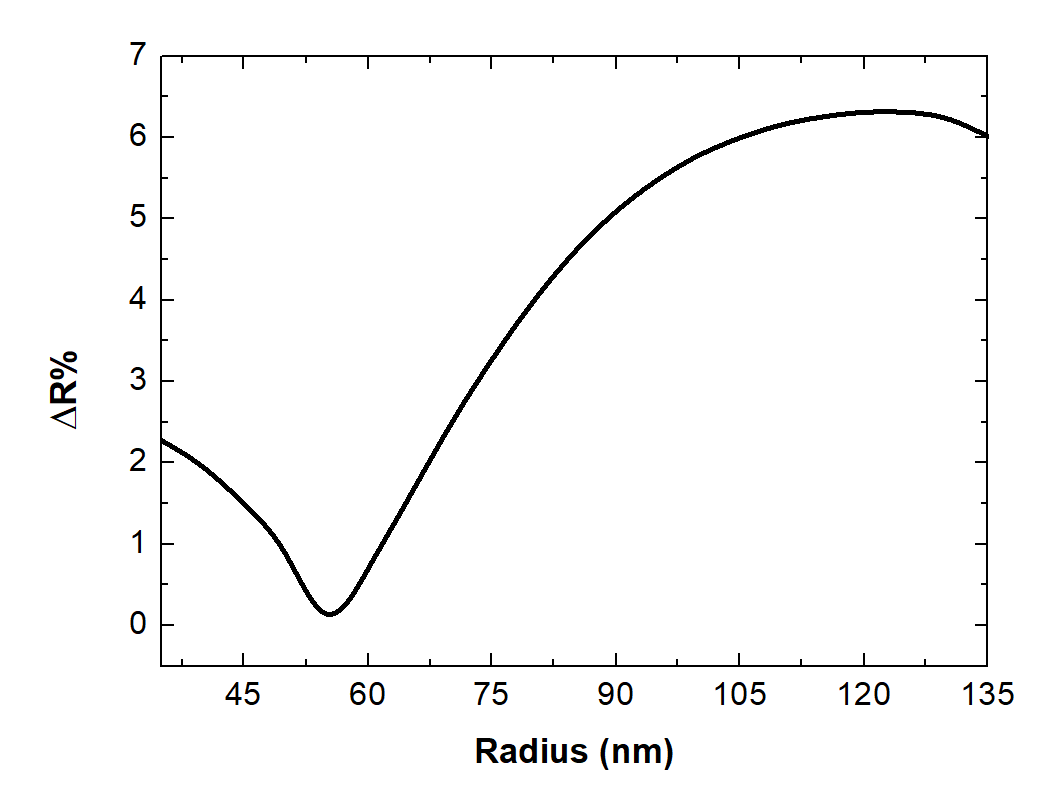


**Figure 17S**: Differential reflectance between RCP and LCP light as a function of the radius (R) of the disk-shaped meta-atoms.


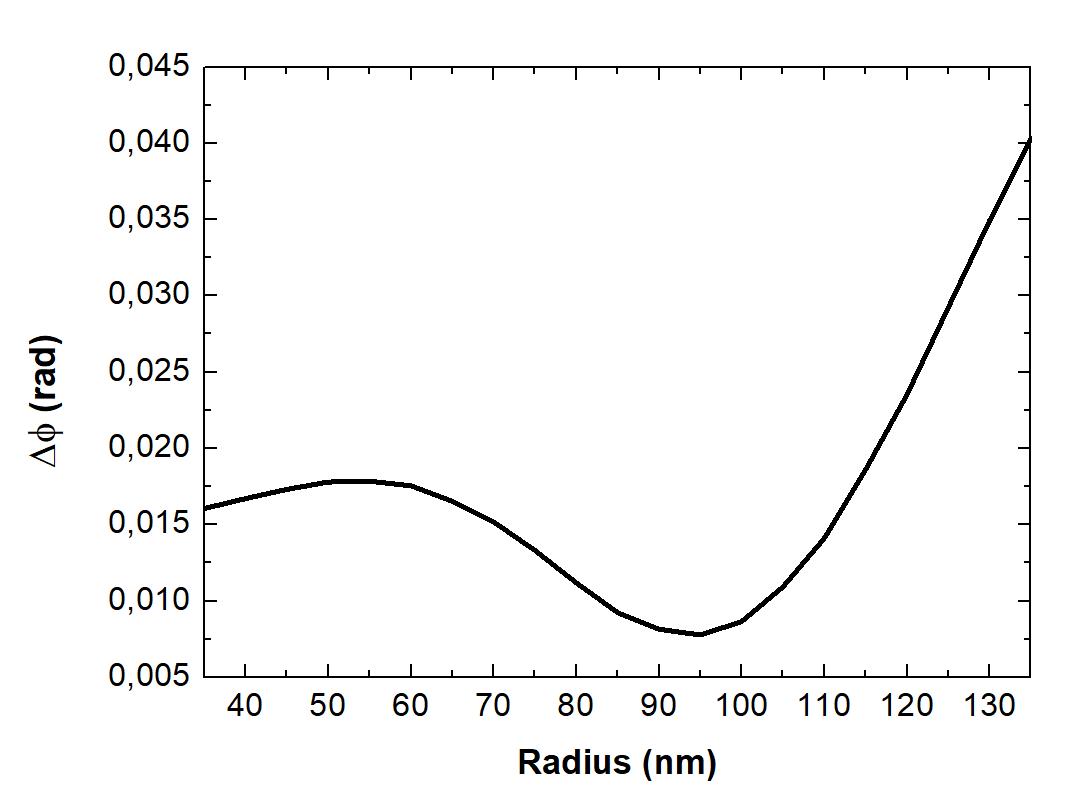


**Figure 18S**: Differential phase between RCP and LCP light as a function of the radius (R) of the disk-shaped meta-atoms.

In Figures 19S, 20S and 21S, we report the reflectance spectrum and the differential spectra of reflectance and phase for LCP and RCP light, varying the dielectric thickness while keeping the following parameters fixed: R = 0.12 𝜇𝑚, t_m_ = 0.012 𝜇𝑚, 𝑃 = 0.28 𝜇𝑚, and 𝜆 = 0.9 𝜇𝑚.

**Figure 19S:** Comparison of the reflectance spectra for the simulated MIM GT meatsurface with RCP (blue line) and LCP (red line) lights.


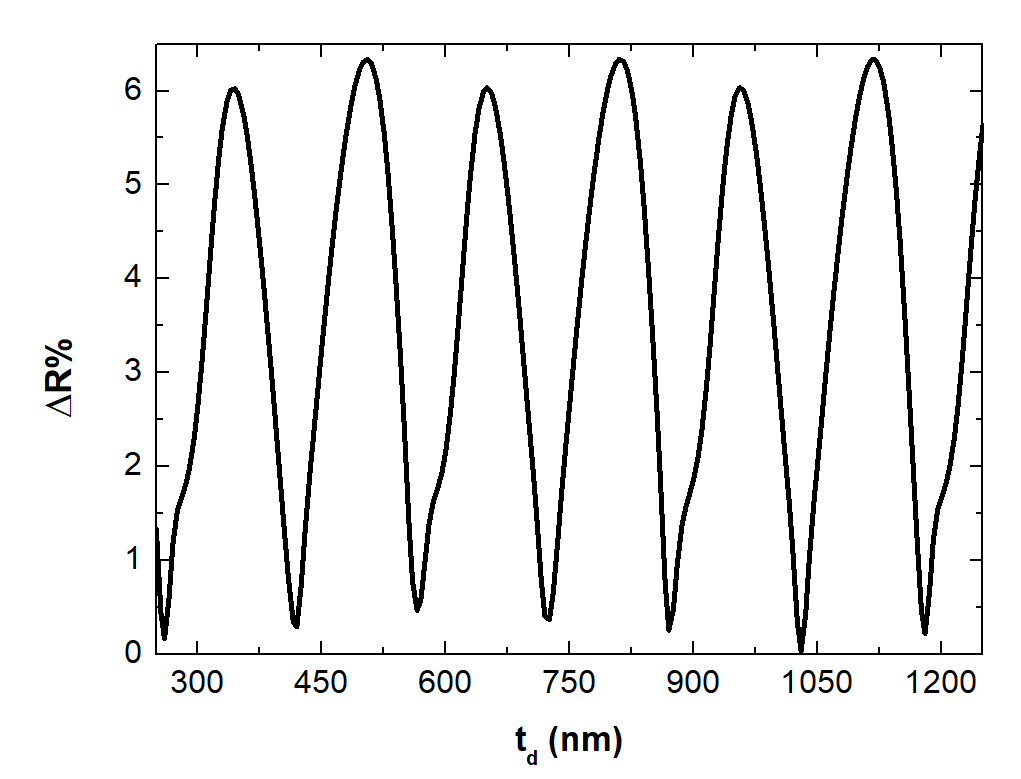


**Figure 20S**: Differential reflectance at 𝜆 = 0.9 𝜇𝑚 between RCP and LCP light as a function of the thickness (t_d_) of the dielectric spacer.


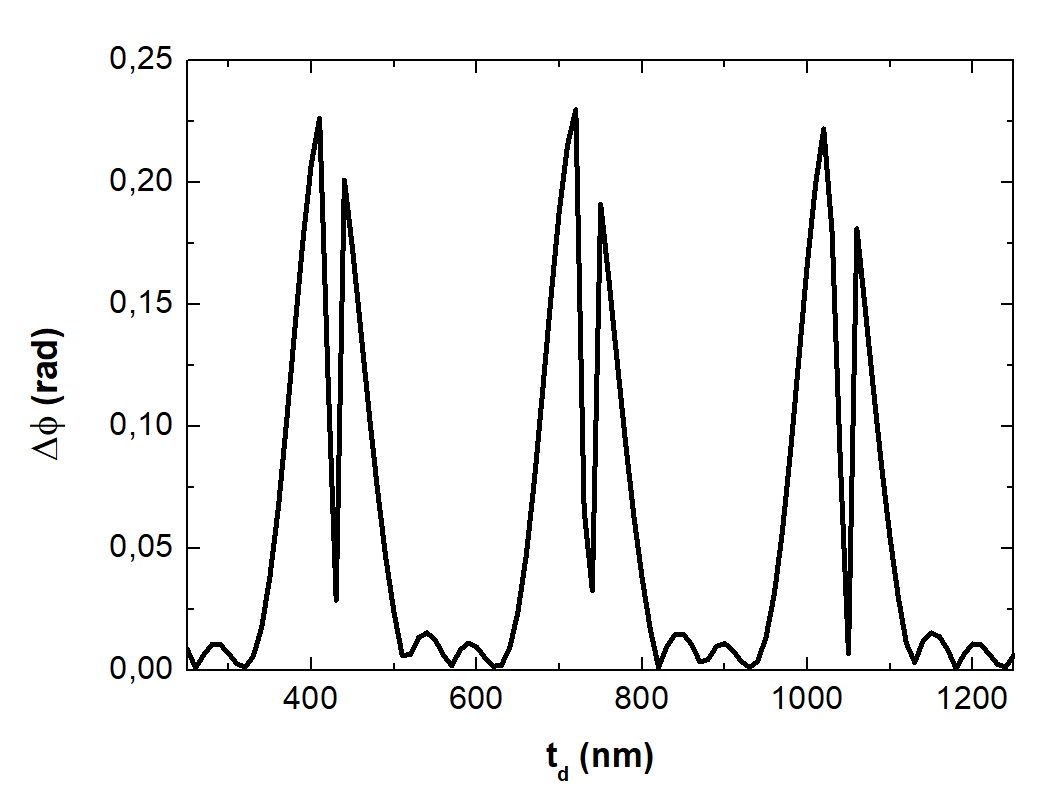


**Figure 21S**: Differential phase at 𝜆 = 0.9 𝜇𝑚 between RCP and LCP light as a function of the thickness (t_d_) of the dielectric spacer.

In Figures 22S and 23S, we report the differential spectra of reflectance and phase for LCP and RCP light, varying the period while keeping the following parameters fixed: R = 0.12 𝜇𝑚, t_m_ = 0.012 𝜇𝑚, t_d_ = 0.5 𝜇𝑚, and 𝜆 = 0.9𝜇𝑚.


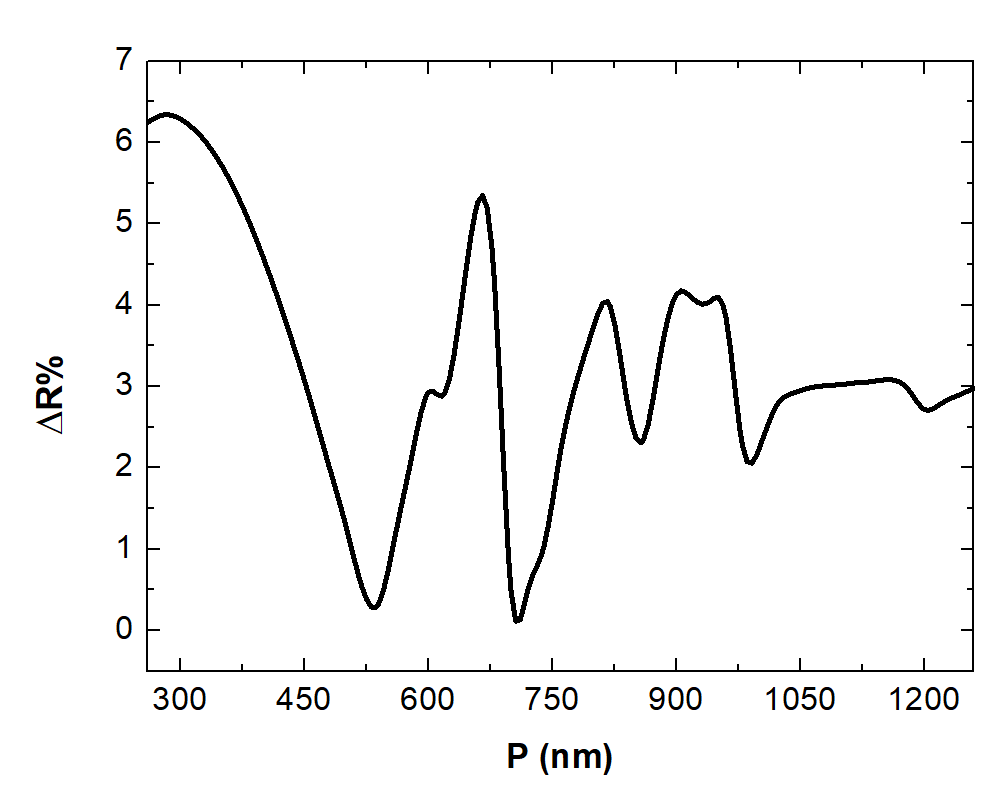


**Figure 22S**: Differential reflectance between RCP and LCP light as a function of the period (P) of the metasurface.


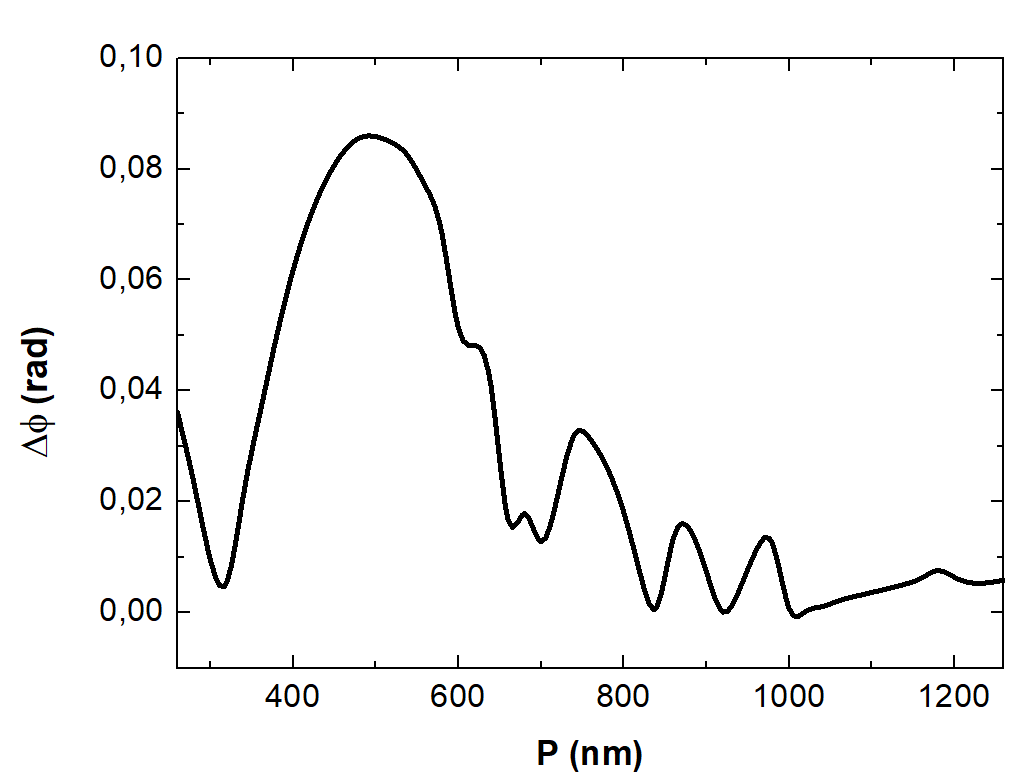


**Figure 23S**: Differential phase between RCP and LCP light as a function of the period (P) of the metasurface.

**l. Surface Lattice Resonances**

The diffraction condition for a grating is described by Equation (17):

$(n_{sub,sup})\sin\left( Ө_{m} \right)+n_{\sup}\sin\left( Ө_{inc} \right)=\frac{\lambda_{0}m}{P}$ (17)

In this expression, *m* is an integer representing the diffraction order. $n_{\mathrm{sub}}$ and $n_{\sup}$ denote the refractive indexes of the upper and lower dielectric, $Ө_{inc}$ and $Ө_{m}$ corresponds to the propagation angle of the incident and of the diffracted beam. In the first addend, we need to use *n_sup_* if the output wave is diffracted in the half space of the incoming beam, or *n_sub_* if it is diffracted into the lower dielectric space.

At normal incidence, the grating equation simplifies to:
$P=\frac{\lambda_{0}n}{m}$ (18)

where $n$ represents the refractive index of the medium in which diffraction is considered, either $n_{\sup}$ for diffraction occurring in the superstrate, or $n_{\mathrm{sub}}$ for diffraction within the substrate.

The condition of Wood’s anomaly is given by Equation (19) in the case of normal incidence:

$P=\frac{\lambda_{0}\left( \frac{\varepsilon_{sub}\varepsilon_{met}}{\varepsilon_{sub}+\varepsilon_{met}} \right)^{\frac{1}{2}}}{m}$ (19)

where $\varepsilon_{sub}$ and $\varepsilon_{met}$ are the permittivities of the dielectric and the metal, respectively.

Surface Lattice Resonances (SLRs) arise from the coupling between localized surface plasmon resonances (LSPRs) of individual metallic nanostructures and the diffracted orders of the periodic array, as defined by the grating condition above. When a diffracted order becomes grazing (i.e., satisfies either grating condition or the Wood’s anomaly condition), the interaction between the scattered light and the array’s periodicity gives rise to collective modes, SLRs. These resonances are highly sensitive to both the lattice period and the surrounding refractive index and are critically dependent on satisfying conditions such as Equations (18) and (19).

We report the electric field distribution of SLRs in a metasurface composed of disk-shaped meta-atoms arranged in a square lattice with a diagonal periodicity of P$\sqrt{\mathbf{2}}$.


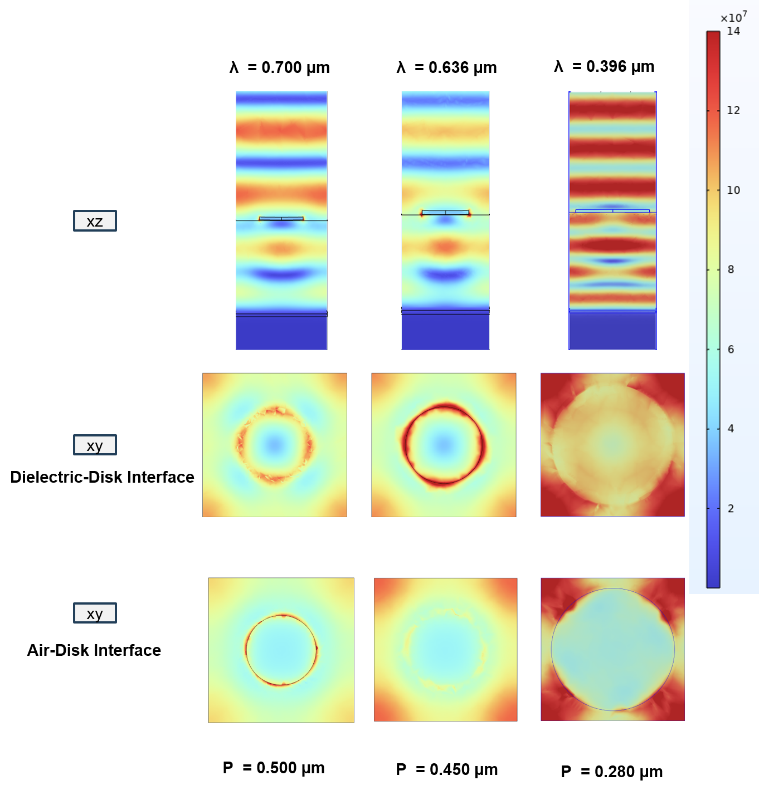


**Figure 24S**: Simulated electric field intensity (|E|) distributions at the resonant wavelengths for metasurfaces with disk-shaped meta-atoms and periods P = 0.500 μm, P =0.450 μm, and P =0.280 μm. Top row: XZ-plane cross-sections. Bottom row: XY-plane field maps at the disk air-disk interface. The results illustrate the emergence and suppression of surface lattice resonances as a function of the array period.

We also report the surface wave anomaly predicted by Wood’s equation for metasurfaces with P = 0.500 μm and P = 0.450 μm. For these periods, surface wave anomaly is observed within the considered spectral range.


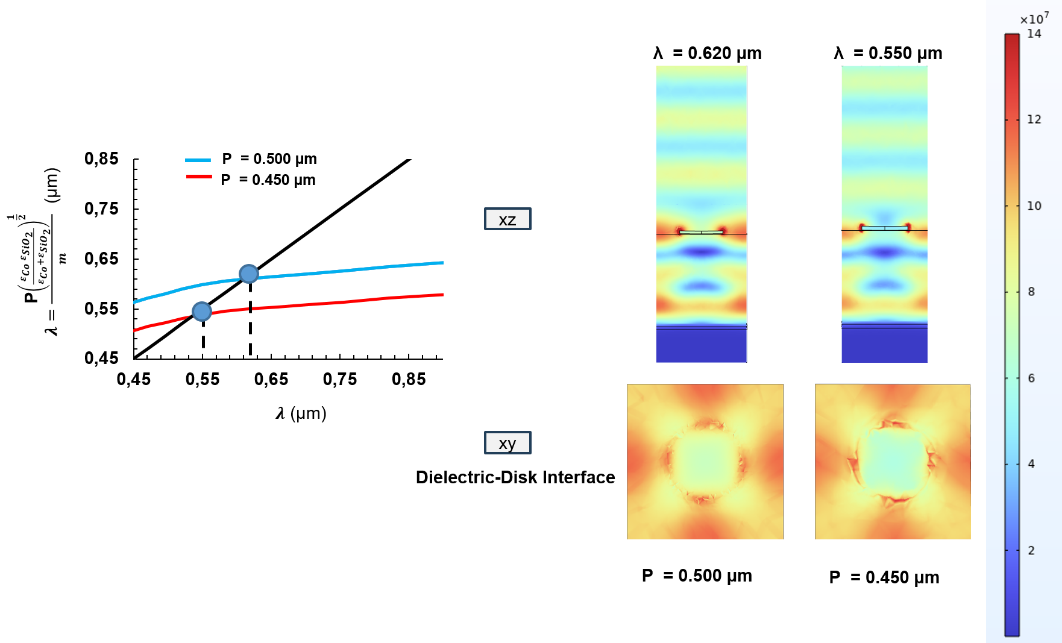


**Figure 25S**: Figure 25S: Simulated electric field intensity distributions (|E|) for metasurfaces with P = 0.500 μm and P = 0.450μm, showing surface wave anomalies associated with Wood’s condition.

Top: XZ-plane cross-sections reveal enhanced field confinement at the disk edges and interface. Bottom: XY-plane maps show lateral coupling patterns consistent with Surface Lattice Resonances.

To complement these field distribution studies and further investigate the fundamental interactions between meta-atoms without cavity and in a homogenous medium (air), we report the absorption spectrum of an infinite array of Co disk shape meta-atoms with period of 500 nm in air.

**Figure 26S**: Correlation between the simulated absorption spectrum and the electric field intensity distributions for an array of Co disks in air.

The absorption spectrum exhibits two distinct regimes, with an array period of 0.5 μm determining the wavelength where the critical transition between the two regimes occurs.

For wavelengths longer than the array period (λ > 0.5 μm), the spectral response is characterized by a broad and smooth absorption band centered near 0.7 μm. This feature corresponds to the LSPR of the individual Co nanodisks. In this regime, the incident wavelength exceeds the periodicity of the array, suppressing higher-order diffraction and preventing collective lattice effects. Consequently, the optical response is dominated by the behaviour of isolated, strongly absorbing resonators. The associated near-field distribution at λ = 0.7 μm corroborates this interpretation: the electric field is highly localized around the disk perimeters, forming intense near-field hotspots that are characteristic of dipolar LSPR excitation.

A qualitatively different optical behaviour emerges for shorter wavelengths (λ ≤ 0.5 μm). In this region, the absorption spectrum evolves from a broad continuum into a sequence of sharp, narrowly spaced spectral modulations observed between 0.4 μm and 0.5 μm. The abrupt onset of these fine spectral features coinciding with the array period is indicative of a SLR (Equation (18)), corresponding to the condition where a diffracted order becomes tangent to the array plane. This marks the transition from localized to collective plasmonic behavior. The resulting spectral modulations originate from the interference between the broad, localized LSPR and the narrow, delocalized diffractive modes supported by the periodic lattice.

**m.** Nickel Based GT Metasurface

We also perform a simulation with the same geometrical parameters of the case reported ion Figure 4 in the paper, but we replaced Co disks with Ni disks. We described Ni using dielectric permittivity diagonal terms from Johnson and Christy [7] and out off diagonal terms from Khrinchik [6]. This allows for a direct comparison between the two systems under equivalent conditions, isolating the influence of the material’s intrinsic magneto-optical properties.

The results of this simulation are summarized in Figures 27S(a) and 27S(b), which present the corresponding reflectance spectra and magneto-optical observables for the Ni-based GT metasurface. More precisely, Figure 27S(a) shows the simulated reflectance spectra for RCP and LCP light, while Figure 27S(b) displays the associated magnetic circular dichroism (ΔA%) and the phase delay difference (Δϕ) between RCP and LCP components as a function of wavelength.

**Figure 27S**: (a) Simulated reflectance spectra for RCP and LCP light interacting with the Ni-based GT metasurface. (b) Corresponding magnetic circular dichroism (ΔA%) and phase delay difference (Δϕ) between RCP and LCP components as a function of wavelength.

When comparing these results with the Co-based system shown in Figure 4 of the paper, the main spectral features occur at nearly the same wavelengths, confirming that the resonant behavior is primarily governed by the metasurface geometry. However, notable differences emerge in the magnitude and shape of the responses: Co-based metasurface exhibits broader reflectance dips and a more pronounced splitting between the RCP and LCP spectra. Likewise, the phase difference between the two circular polarizations is stronger in the Co-based case under the same external magnetic field amplitude.

These contrasting behaviours can be directly attributed to the difference in the intrinsic magneto-optical coupling strength of the two materials, which is quantified by the complex magneto-optical constant $Q$.

In magneto-optical materials, the complex quantity

$Q=\frac{i\varepsilon_{xy}}{\varepsilon_{xx}}$ (20)

represents the magneto-optical constant, which quantifies the coupling between the material optical and magnetic responses. Here, $\varepsilon_{xx}$ is the diagonal component of the dielectric tensor, describing the ordinary optical response, while $\varepsilon_{xy}$ is the off-diagonal component induced by magnetization, responsible for magneto-optical effects such as the Kerr and Faraday rotations.

The magnitude ∣*Q*∣ therefore serves as a figure of merit for the strength of magneto-optical activity in the material. Larger values of ∣*Q*∣ correspond to stronger magneto-optical interactions and enhanced polarization rotation in reflected or transmitted light.

Figure 28S reports a comparison between ∣*Q*∣ for Co and Ni.


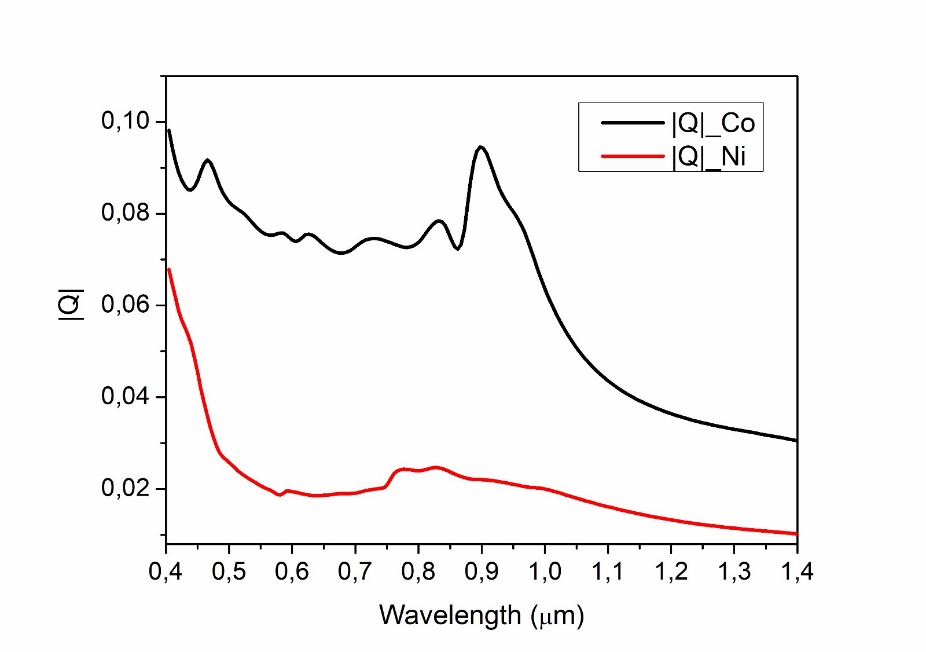


Figure 28S: Comparison of the wavelength-dependent magneto-optical figure of merit ∣*Q*∣ for Co (black) and Ni (red).

In the plotted comparison, Co exhibits significantly higher ∣*Q*∣ values across the spectral range (0.4 μm - 1.4 μm) than Ni. This indicates that Co possesses a stronger magneto-optical response.

**References**

[1] A. Gabbani, G. Petrucci, and F. Pineider, “Magneto-optical methods for magnetoplasmonics in noble metal nanostructures,” *J. Appl. Phys.*, vol. 129, no. 21, 2021.

[2] A. Gabbani *et al.*, “High magnetic field magneto-optics on plasmonic silica-embedded silver nanoparticles,” *J. Phys. Chem. C*, vol. 126, no. 4, pp. 1939–1945, 2022.

[3] B. Sepúlveda, J. B. González-Díaz, A. García-Martín, L. M. Lechuga, and G. Armelles, “Plasmon-induced magneto-optical activity in nanosized gold disks,” *Phys. Rev. Lett.*, vol. 104, no. 14, p. 147401, 2010.

[4] N. Maccaferri *et al.*, “Tuning the Magneto-Optical Response of Nanosize Ferromagnetic Ni Disks Using the Phase of Localized Plasmons,” *Phys. Rev. Lett.*, vol. 111, no. 16, p. 167401, 2013.

[5] J. B. González-Díaz *et al.*, “Plasmonic Au/Co/Au nanosandwiches with enhanced magneto-optical activity,” 2008.

[6] G. S. Krinchik and V. A. Artem’Ev, “Magneto-optical properties of Ni, Co, and Fe in the ultraviolet, visible, and infrared parts of the spectrum,” *Sov. Phys. JETP*, vol. 26, no. 6, pp. 1080–1085, 1968.

[7] P. B. Johnson and R. W. Christy, “Optical constants of transition metals: Ti, v, cr, mn, fe, co, ni, and pd,” *Phys. Rev. B*, vol. 9, no. 12, p. 5056, 1974.
